# Immunogenic cell death unlocks the potential for combined radiation and immunotherapy

**DOI:** 10.1073/pnas.2509875122

**Published:** 2025-11-26

**Authors:** Somiya Rauf, Alexandra Smirnova, Andres Chang, Yuan Liu, Yi Jiang

**Affiliations:** ^a^Department of Mathematics and Statistics, Georgia State University, Atlanta, GA 30303; ^b^Department of Hematology and Medical Oncology, Winship Cancer Institute of Emory University, Atlanta, GA 30322; ^c^Key State Laboratory of Pharmaceutical Biotechnology, Nanjing University School of Life Sciences, Nanjing, Jiangsu 210046, China

**Keywords:** immunogenic cell death, immune checkpoint inhibitor, SIRP*α*-CD47 blockade, radiotherapy, phagocytosis

## Abstract

Immunogenic cell death (ICD) enhances antitumor immunity by releasing tumor-associated antigens and activating the antitumor immune system response. Here, we develop a mathematical model to quantify the role of ICD in optimizing the efficacy of combined radiotherapy (RT) and macrophage-based immunotherapy. Using preclinical murine data targeting the SIRPα-CD47 checkpoint, we show that RT alone induces minimal ICD, whereas disrupting the SIRPα-CD47 axis significantly enhances both phagocytosis and systemic immune activation. Our model predicts an optimal RT dose (6 to 8 Gy) for maximizing ICD, a dose-dependent abscopal effect, and a hierarchy of treatment efficacy, with SIRPα-knockout macrophages exhibiting the strongest tumoricidal activity. These findings provide a quantitative framework for designing more effective combination therapies, leveraging ICD to enhance immune checkpoint inhibition and radiotherapy synergy.

## Significance

Immunogenic cell death (ICD) is emerging as a critical mechanism that bridges local cancer therapy with systemic immune responses. This study provides a quantitative framework that integrates ICD, radiation therapy (RT), and macrophage-based immunotherapy targeting the SIRPα-CD47 checkpoint. Using a rigorously calibrated mathematical model informed by in vivo data, we identify an optimal RT dose range (6 to 8 Gy) that maximizes ICD and reveal that SIRPα-CD47 checkpoint inhibition transforms radiation-induced tumor cell death into a potent systemic immune stimulus. Our model predicts abscopal effects, quantifies treatment efficacy across tumor types, and ranks therapeutic strategies by their ability to enhance phagocytosis and immune activation. These insights offer a rational basis for designing combination therapies to improve clinical outcomes.

Immunotherapy that harnesses the immune system to eliminate malignant cells has transformed cancer treatment over the past two decades (1). Immunogenic cell death (ICD) plays a critical role in bridging local cancer therapy with systemic immune responses (2). ICD involves changes in the cell surface as well as the release of soluble mediators, such as the externalization of calreticulin and the release of adenosine triphosphate (ATP) and HMGB1 (3–5). These signals add to other damage-associated molecular patterns (DAMPs) released by stressed or injured cells, signal antigen-presenting cells, including dendritic cells and macrophages, to activate tumor-specific cytotoxic T cells (6), setting off an antitumor immune response and additional ICD. This positive feedback loop not only eliminates the dying tumor cells but also targets therapy-resistant cancer stem cells, making ICD a critical mechanism underlying the efficacy of many anticancer therapies (6, 7).

Radiotherapy (RT) not only causes direct DNA damage to tumor cells but can also trigger ICD (8) in a dose-dependent manner (9). However, RT alone often produces limited immunogenicity, particularly in large or poorly immunogenic tumors, because it simultaneously damages immune cells and fosters an immunosuppressive microenvironment (10, 11). Optimizing RT to maximize ICD while minimizing immune suppression and preserving immune function remains a critical challenge.

Macrophages are central regulators of the tumor microenvironment, with phenotypic plasticity that allows polarization toward either tumoricidal (M1) or tumor-promoting (M2) states (12–14). A major checkpoint regulating macrophage phagocytosis is the SIRPα-CD47 axis. CD47, overexpressed by many tumors (15), delivers a “don’t eat me” signal by binding to SIRPα on macrophages, suppressing phagocytosis (16). Blocking this checkpoint through antibodies, fusion proteins, or genetic deletion (e.g., SIRPα-deletion) has been shown in preclinical models to enhance phagocytosis, promote M1 polarization, and improve tumor clearance (17–21).

Despite promising preclinical results supporting the therapeutic potential of SIRPα-CD47 blockade (22–25), its clinical translation remains difficult. A key challenge lies in the incomplete understanding of the interaction between this macrophage-based immunotherapy and RT. In particular, how ICD, radiation dose, and macrophage checkpoint inhibition collectively determine treatment efficacy, both locally and systemically, remains an open question. While mathematical models have provided valuable insights into tumor–immune dynamics and therapy (26–30), few have attempted to integrate ICD with both RT and macrophage-based immunotherapy.

Here, we address the lack of a quantitative framework to explain and predict how ICD mediates the synergy between RT and macrophage-based immunotherapies targeting the SIRPα-CD47 axis. No single mathematical model can incorporate every detail of tumor growth in vivo, tumor microenvironment (TME), immunotherapy, and RT. However, such exhaustive complexity is not necessary for a model to be useful or predictive. Parsimony is critical to ensure interpretability, identifiability, and reliable calibration with the available data. Our model integrates radiotherapy, ICD, and the SIRPα-CD47 signaling, and is calibrated using in vivo data. We find that ICD is minimal under RT alone but is substantially amplified when SIRPα-CD47 interactions are disrupted. This synergy predicts an optimal RT dose range (6 to 8 Gy), ranks therapeutic strategies by their ability to enhance phagocytosis, and demonstrates conditions that enable abscopal effects. Together, these findings provide testable mechanisms of therapeutic synergy, offer a rational basis for optimizing combination therapies and guiding future translational studies.

## Results

### A Mathematical Model for In Vivo Tumor Growth Under RT and Macrophage-Based Immunotherapy.

**Fig. 1.**
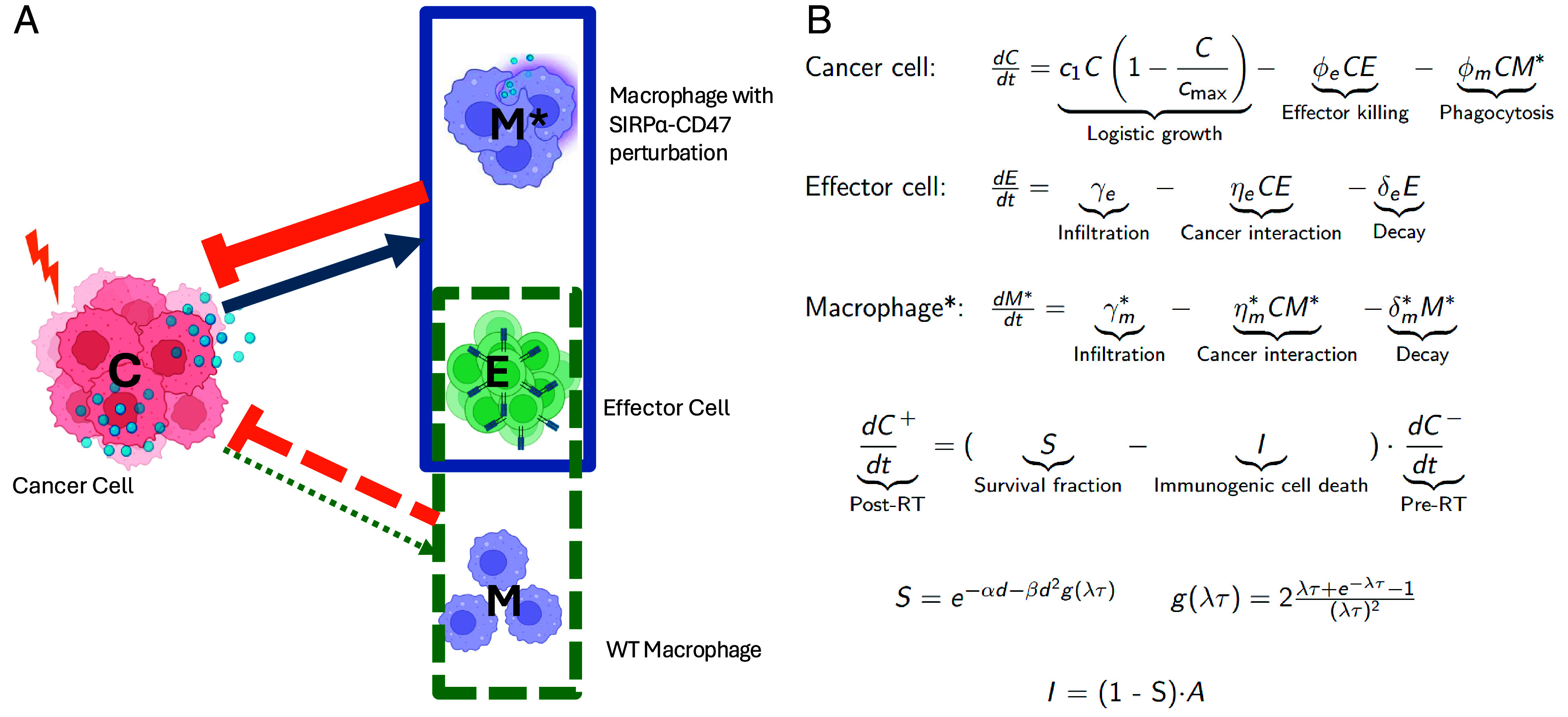
Overview of the mathematical model: (*A*) Radiation induces minimal activation of the immunogenic cell death (ICD) pathway (dashed green arrow) with the wild-type (WT) macrophages. In contrast, macrophages with disrupted SIRPα-CD47 signaling respond to RT-induced DAMPs, activating ICD (solid blue arrow) and thereby enhancing tumor elimination (solid orange hammerhead). (*B*) Model equations describe the dynamics of cancer cells (C), effector cells (E), and either WT macrophages (M) or SIRP$\alpha^{-}$/SIRPα/CD47-treated macrophage ($M^{*}$). Postradiation cancer cells ($C^{+}$) represent the surviving fraction of pre-RT tumor cells ($C^{-}$). Survival is governed by the linear-quadratic (LQ) dose-dependent survival fraction (S), parameterized by radio-sensitivity coefficients α, β, repair rate λ, and RT delivery time τ. ICD (I) is proportional to radiation-induced tumor cell damage (1−S) and is scaled by the immune activation parameter A. Full details are provided in *Methods* and *SI Appendix*.

Our model considers three populations (cancer cells, effector cells, and macrophages) and four ways of cancer cell death (effector killing, phagocytosis by macrophage, radiation cell death, and immunogenic cell death), as illustrated in Fig. 1. Radiation-damaged cells activate the ICD pathway by activating the effector cells that kill more cancer cells (blue arrow in Fig. 1*A*). Macrophages with disrupted CD47/SIRPα binding promote a highly proinflammatory TME and enhance ICD (orange arrows). Briefly, tumor growth follows a logistic dynamics and is either inhibited by effector cells or phagocytic macrophages. Effector cells infiltrate the tumor, become exhausted with reduced polyfunctionality, or are cleared at a rate that we assume to be constant. Similarly, macrophages infiltrate, become exhausted from phagocytosing tumor cells or are cleared at a rate assumed to be constant. RT reduces tumor cell survival in a dose-dependent manner, with immunogenic cell death proportional to the RT-induced cell death (Fig. 1*B*).

We calibrate the model using tumor growth data from a preclinical study using treatment-resistant colorectal adenocarcinoma (MC38) in WT (C57BL/6) and SIRP$\alpha^{-/-}$ mice, with and without local irradiation. Unless otherwise noted, all experimental data used in this paper were previously published by Bian, et al. (22). This calibration is done in four steps of increasing complexity. We first find the parameters of tumor growth in WT mice (*SI Appendix*, Table S3). We fix these parameters when we calibrate RT-related parameters of tumor growth in WT with RT (*SI Appendix*, Table S5), and SIRP$\alpha^{-}$ macrophage related parameters using tumor growth data in SIRP$\alpha^{-/-}$ mice without RT (*SI Appendix*, Table S4). We then calibrate for the last parameters for tumor growth in SIRP$\alpha^{-/-}$ mice with RT (Table 1). The calibration processing constrains possible explanations of experimental data, and the calibrated model allows for predictions. The prediction results reported below are calculated using the calibrated model without any free parameters. An important consideration is that in vivo tumor growth data, measured in volume, includes both cancer cells and infiltrated immune cells.

**Table 1.** ICD and phagocytosis values for MC38 tumors across different SIRP*α*-CD47 treatments

Mouse model & treatment (data source)	Day	Tumor volume (mm^3^)	Dose (Gy)	*A*	*I*	$\phi_{m}$ ($\times 10^{-9}$)
WT (22)	8	0 to 100	4	0	0	0.357
			8	0	0	
			15	0	0	
	12	100 to 400	4	0	0	0.357
			8	0	0	
			15	0	0	
	14	400 to 600	4	0	0	0.357
			8	0	0	
			15	0	0	
SIRP$\alpha^{-/-}$ (22)	8	0 to 100	4	34.88	1.44	907
			8	21.07	1.76	
			15	9.58	1.47	
	12	100 to 400	4	27.70	1.15	907
			8	15.32	1.28	
			15	7.66	1.17	
	14	400 to 600	4	11.34	0.47	907
			8	8.25	0.69	
			15	5.31	0.82	
WT with SIRPα-KO (22)	12 & 14	150 to 300	8	8.24 &19.9	0.69 &1.67	44.3
WT with anti-SIRPα (25)	9	100 to 300	12	0.98	0.30	43.4

### RT in WT Mice Fails to Induce ICD.

**Fig. 2.**
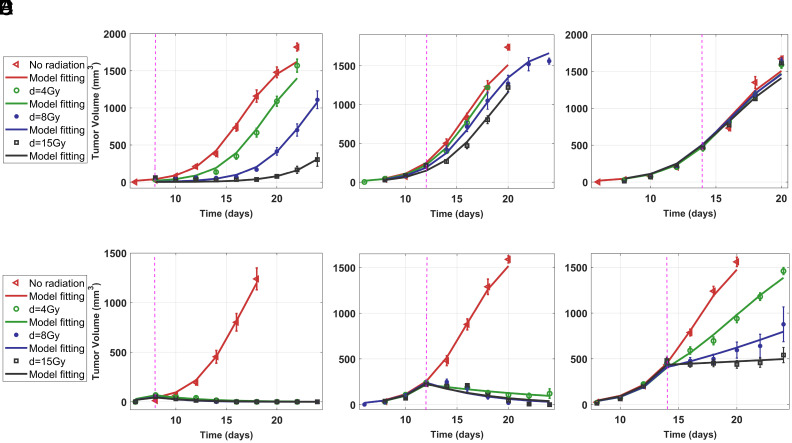
RT is ineffective for large tumors in WT mice but significantly reduces tumor growth in SIRP$\alpha^{-/-}$ mice due to ICD: (*A*–*C*) In WT mice, small tumors (0 to 100 mm^3^) respond well to higher doses of RT (*A*), while medium-sized tumors (100 to 400 mm^3^) exhibit only a modest reduction even with large doses (*B*). Large tumors (400 to 600 mm^3^) show minimal reduction in size (*C*). (*D*–*F*) In SIRP$\alpha^{-/-}$ mice, RT effectively eliminates small tumors (*E*), remains effective for medium-sized tumors, (*E*), and offers significant reduction for large tumors (*F*) depending on radiation dose. Experimental data from ref. 22.

The model effectively replicates tumor growth data in WT mice with RT of various doses, where MC38 cells ($5\times 10^{5}$) were subcutaneously engrafted into the right flank of WT mice and irradiation was delivered on day 8, 12, and 14, corresponding to tumor volumes of 0 to 100 mm^3^ (small), 100 to 400 mm^3^ (medium), and 400 to 600 mm^3^ (large), respectively (data from ref. 22, Fig. 2*A*–*C*). RT delivered on later days and larger tumors results in less tumor reduction. For these tumor sizes and RT doses, immunogenic cell death values (I=(1−S)A) remain zero or negligible compared to ICD values when the SIRPα-CD47 pathway is disrupted (Table 1). These results suggest that in WT mice treated with RT, there is no impact of ICD, even for small tumors subjected to high-dose irradiation.

### ICD in SIRP*α*^−/−^ Mouse Depends on Tumor Size and Radiation Dose.

In SIRP$\alpha^{-/-}$ mice, significant tumor reduction is evident following RT, even in large tumors (Fig. 2*D*–*F*). This increased tumor reduction can be attributed to ICD, which is influenced by the interplay between RT cancer cell damage and effector cell activation. The corresponding ICD values for small, medium, and large tumors at RT doses of 4, 8, and 15 Gy are summarized in Table 1.

While the values for ICD activation parameter A and ICD I vary across different tumor volumes and RT doses (Table 1), an interesting trend emerges: ICD consistently peaks at an RT dose of 8 Gy for all tumor sizes. This observation can be explained by the fundamental mechanism of ICD. Radiation-induced cancer cell damage (1−S) increases with dose; however, ICD activation, A, decreases at higher doses (Table 1) due to RT-induced damage to immune cells, as observed in tumor control probability (TCP) and normal tissue complication probability (NTCP) (31). The interplay between these two opposing effects results in a biphasic dependence of ICD on radiation dose. Notably, previous studies have also shown that intermediate radiation doses provide optimal treatment effects in mice (32).

**Fig. 3.**
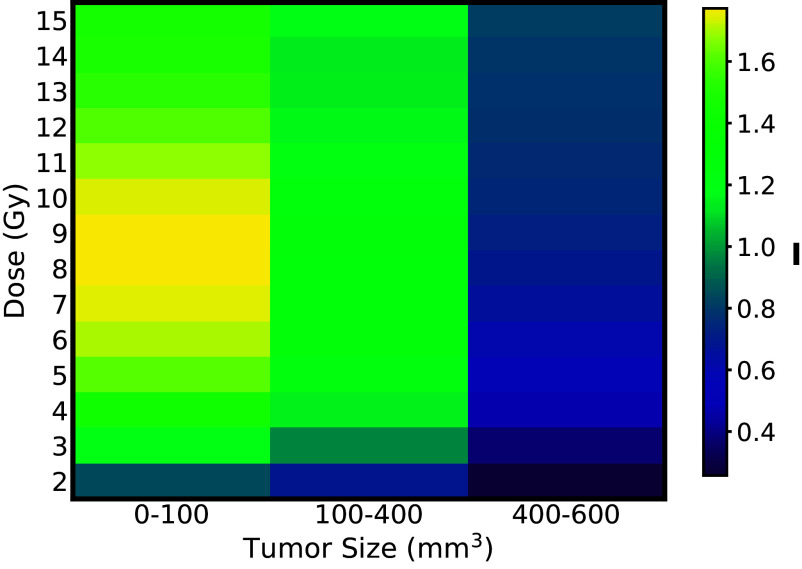
Optimizing RT doses to enhance ICD in SIRP$\alpha^{-/-}$ mice: Immunogenic death induced (I) by RT in the SIRP$\alpha^{-/-}$ mouse model. The heat-map highlights high immune activation for intermediate doses for small and intermediate tumor volumes. For large tumors, the medium and high doses of RT provide better results.

We explore the ICD (I) values across different tumor volumes and radiation doses to determine the effectiveness of RT in inducing ICD (Fig. 3). This result is calculated by the spline interpolation of the immune activation parameter A in Table 1. Notably, in small and medium-sized tumors, a progressive increase in immune activation is observed with increasing doses up to a threshold of 6 Gy. This observation suggests that the optimal RT dose is close to 6 Gy for smaller tumors (<400 mm^3^). For large tumors, despite the increasing radiation doses, the rate of immune activation increase is more gradual till 10 Gy, suggesting that larger tumors may pose challenges in eliciting a strong immune-mediated antitumor effect through radiation alone. This underscores the importance of considering tumor burden when designing radiation doses for optimal ICD and highlights the potential need for combined therapies to overcome immune resistance in larger tumors.

The sensitivity analysis of LQ model parameters in WT and SIRP$\alpha^{-/-}$ mice, focusing on different tumor sizes, reveals that these RT parameters have minimal impact on tumor response in WT mice, while a more pronounced effect is observed in SIRP$\alpha^{-/-}$ mice (*SI Appendix*, Fig. S4).

### Combined with RT, SIRPα-Deficient Macrophages Effectively Reduce Tumor Growth via ICD.

**Fig. 4.**
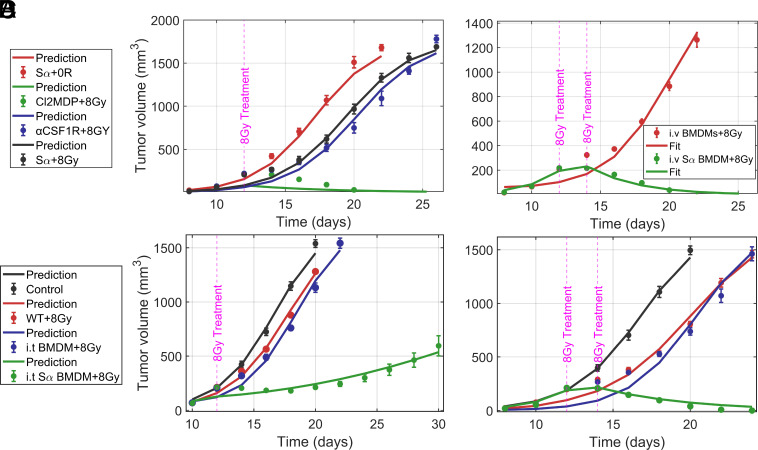
SIRPα-deficient macrophages reduce tumor growth in WT mice via ICD. (*A*) Mathematical model predicts no tumor killing in SIRP$\alpha^{-/-}$ mice with RT induced ICD when macrophages are depleted. (*B*) The mathematical model captures tumor elimination by twice i.v. injection of SIRPα-deficient macrophages with RT of 8 Gy. (*C* and *D*) The model predicts that single and dual intratumoral (i.t.) treatments with BMDM and SIRP$\alpha^{-}$ macrophages in WT mice with 8 Gy irradiation yield comparable tumor elimination as in i.v. injection case. Experimental data from ref. 22.

In SIRP$\alpha^{-/-}$ mice, tumor responsiveness to RT is contingent upon the presence of SIRP$\alpha^{-}$ macrophages (22). Depletion of intratumoral (i.t.) macrophages using either Cl2MDA liposomes or an antibody against the CSF1 receptor (αCSF1R) abolishes the efficacy of RT in SIRP$\alpha^{-/-}$ mice (22). Our model accurately predicted this effect without any fitting parameters (Fig. 4*A*).

SIRPα-deficient macrophages can have complete antitumor treatment effects in WT mice when injected intravenously (i.v.) accompanied by RT (22) (Fig. 4*B*). We allow only two free parameters for fitting these experimental data: immune activation A and macrophage phagocytosis rate $\phi_{m}$. We find that for the first i.v. injection on day 12 with 8 Gy RT, $A_{d1}=8.247$ (corresponding to I=0.69), and the second i.v. injection with RT on day 14, $A_{d2}=19.95$ (I=1.67). The immune activation for the second treatment is significantly higher than for the first, suggesting that immune priming is at work. Macrophage phagocytosis rate $\phi_{m}=4.43\times 10^{-8}$ remains the same for both treatments.

Next, using $A_{d1}$, $A_{d2}$, and $\phi_{m}$ values from i.v. injection experiments, we predict the tumor response when WT mice receive intratumoral (i.t.) injection of WT (SIRP$\alpha^{+}$ bone marrow–derived macrophages (BMDM), and SIRP$\alpha^{-}$ macrophages, in conjunction with RT at 8 Gy (22). The model successfully predicts the reduction in tumor growth with a single i.t. injection using $A_{d1}$ (Fig. 4*C*), and twice i.t. injections using both $A_{d1}$ and $A_{d2}$ (Fig. 4*D*). This confirms that immune activation for i.t. and i.v. injections results in the same values of ICD. Injecting SIRP$\alpha^{-}$ macrophages to WT mice transforms the TME into a state resembling that in SIRP$\alpha^{-/-}$ mice.

#### Model predicts phagocytosis order in SIRP*α*-CD47 checkpoint inhibitions.

**Fig. 5.**
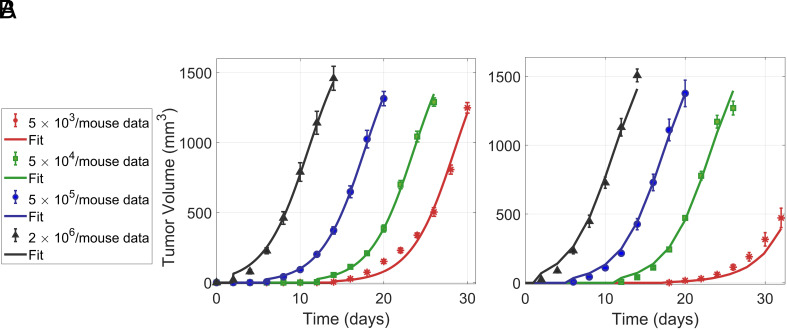
SIRP$\alpha^{-}$macrophages show higher phagocytosis rates than WT: Tumor growth patterns across four tumor cell engraftments in (*A*) WT and (*B*) SIRP$\alpha^{-/-}$ mice, with model fitting (solid lines) and experimental data (dots with error bars), experimental data from ref. 22.

In both WT and SIRP$\alpha^{-/-}$ mice, the rate of tumor growth depends on the number of engrafted MC38 cells: A higher number of cells leads to more rapid tumor growth (22). In WT mice (Fig. 5*A*), we find that a smaller initial tumor size leads to a larger intratumoral effector cell population, which in turn reduces tumor volume. The effector cells are responsible for lowering the tumor volume because macrophage phagocytosis rate is one order of magnitude lower than the reported M1 macrophage phagocytosis rate (33) (*SI Appendix*, Table S1). In addition, we show that tumor growth in WT mice is insensitive to macrophage-related parameters, suggesting that WT macrophages have little impact on the tumor volume change (*SI Appendix*). Moreover, while the growth rates of tumors are similar between WT and SIRP$\alpha^{-/-}$ mice when larger numbers of MC38 cells are engrafted. SIRP$\alpha^{-/-}$ mice exhibit a significantly slower tumor growth rate with a smaller engraftment at $5\times 10^{3}$ cells/mouse (Fig. 5*B*). Our model demonstrates that the phagocytosis rate of SIRP$\alpha^{-}$ macrophages is the primary factor accounting for this variance. Interestingly, this phagocytosis rate does not reduce tumor growth for large engraftments, due to a rapid decrease of macrophages in SIRP$\alpha^{-/-}$ mice (*SI Appendix*, Table S4).

Both anti-CD47 and CD47-KO promote phagocytosis by disabling the “don’t eat me” signal (23). We find that inhibitions of SIRPα-CD47 checkpoint together with RT in WT mice increase the phagocytosis rate by two orders of magnitude (Table 1). This observation aligns with experimental findings (22–25, 34, 35), which report the enhanced phagocytic capability of SIRPα/CD47 treated models. CD47 inhibition combined with RT yields greater tumor clearance than either treatment alone, consistent with enhanced T cell infiltration as described (35).

Interestingly, macrophages in two different anti-SIRPα studies (24, 25), where RT of different doses was applied to tumors of different sizes, show the same order of magnitude of phagocytosis rates. These results also allow us to rank the phagocytosis rates of macrophages with SIRPα-CD47 checkpoint inhibition, in descending order: SIRPα-KO, anti-SIRPα, and anti-CD47. We do not have MC38 tumor growth data with combined CD47-KO and RT in WT mice (Tables 1 and 2). It should be noted that parameter values may vary slightly because $\phi_{m}$ is practically unidentifiable; however, the ranking order remains consistent.

**Table 2.** Summary of treatments in other mouse models and other cell lines: Among the tested cell lines, the KPC cell line exhibited the strongest ICD response, surpassing MC38 and Pan02

Treatment (data ref)	Mice	Cell line	Day	Dose (Gy)	I	$\phi_{m}\left(\times 10^{-8}\right)$
SIRPα deficiency (22)	SIRP$\alpha^{-/-}$	Pan02	12	8	1.29	90.7
SIRPα deficiency (22)	SIRP$\alpha^{-/-}$	KPC	18	8	1.45	90.7
Anti-CD47 (23)	NSG	KP1	10	5	0.25	1.43
Anti-CD47 (23)	NSG	KP1	12	5	0.97	1.43
CD47-KO (23)	NSG	KP1	11, 13	5, 5	0.17, 0.53	74.6
CD47-KO (23)	NSG	KP1	10, 14	5, 5	0.03, 0.44	74.6
Anti-SIRPα (24)	WT	MC38-OVA	12	8	0.27	4.34
Anti-CD47 (24)	WT	MC38-OVA	12	8	0.22	1.43

Anti-CD47 therapy induced a high ICD compared to CD47-KO.

### Model Explains Enhanced Tumor Clearance with Combined RT and SIRP*α*-CD47 Checkpoint Inhibitions in Other Mouse and Tumor Models.

**Fig. 6.**
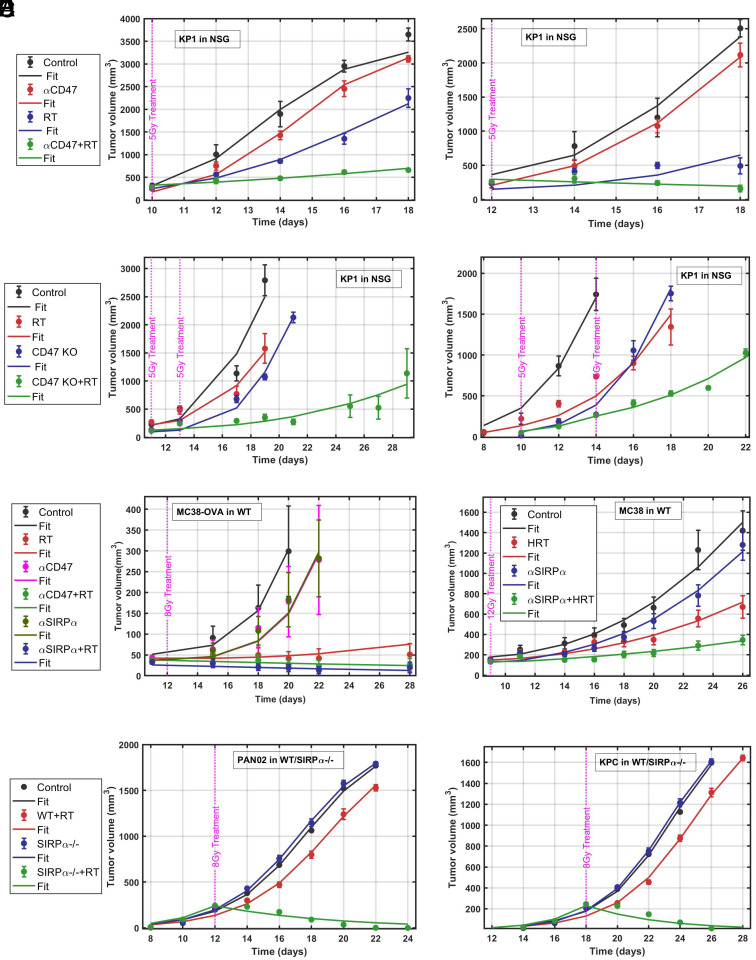
Model explains enhanced tumor clearance with combined SIRPα-CD47 checkpoint inhibitions and RT across various mouse and tumor models: (*A*) KP1 tumors in NSG mice treated with anti-CD47 and RT, based on experimental data from ref. 23. (*B*) KP1 tumors in NSG mice treated with CD47-KO with multiple RT, based on experimental data from ref. 23. (*C*) MC38-OVA tumors in WT mice treated with anti-SIRPα and anti-CD47 and RT, based on experimental data from ref. 24. (*D*) MC38 tumors in WT mice treated with anti-SIRPα and RT, based on experimental data from ref. 25. (*E*) Pan02 tumor growth in WT and SIRP$\alpha^{-/-}$ mice with RT, based on experimental data from ref. 22. (*F*) KPC tumor growth in WT and SIRP$\alpha^{-/-}$ mice with RT, based on experimental data from ref. 22. Model-fitted parameters allow us to compare the macrophage phagocytosis rates and ICD values.

To determine whether our model is useful for only MC38 tumors in WT and SIRP$\alpha^{-/-}$ mice, we apply the model to other tumor and mouse models with combined RT and SIRPα-CD47 checkpoint inhibitions. We use tumor growth data for KP1 small cell lung cancer (SCLC) allografts in immunodeficient NSG mice under RT in combination with CD47 blockade (Fig. 6 *A* and *B*), data from ref. 23, MC38-Ovalbumin (MC38-OVA) tumor in WT mice with RT and anti-CD47 or anti-SIRPα treatment (Fig. 6*C*), data from ref. 24, and MC38 tumor in WT mice with anti-SIRPα and high-dose radiotherapy (HRT) (Fig. 6*D*) experimental data from ref. 25. In addition, we use growth data from pancreatic cancer cell lines Pan01 and KPC in WT and SIRP$\alpha^{-/-}$ mice (Fig. 6 *E* and *F*) (22). Our model is able to fit all tumor growth curves, showcasing its general applicability (Fig. 6). Their phagocytosis rate ($\phi_{m}$) and ICD values (I) are summarized in Table 2. All other parameter values for different cell lines and mouse models are summarized in *SI Appendix*, Tables S6 and S7.

Because of substantial differences in genetic background, immune competency, and tumor microenvironment among various mouse models, direct comparisons of tumor growth across these models are confounded by multiple uncontrolled variables. Attempting to draw meaningful parallels or establish universal growth parameters from such heterogeneous systems often lacks scientific rigor and can lead to misleading conclusions. We therefore only compare within the same tumor cell lines or within the same mouse models.

The ICD value I in MC38 tumors in WT mice with combined RT and anti-CD47 treatment is significantly lower than that in SIRP$\alpha^{-/-}$ mice (Table 2). In addition, the ICD values for MC38 tumors across different SIRPα-CD47 checkpoint inhibitions in combination with RT in WT mice show a clear pattern: Earlier treatment leads to enhanced ICD (Table 1). Treatment of RT with SIRPα-KO achieves the highest ICD, followed in descending order by anti-SIRPα, anti-CD47, CD47-KO treatments, and control (no treatment). A second treatment elicits much higher ICD, suggesting that the immune system could be primed by the first treatment.

In SIRP$\alpha^{-/-}$ mice, immune activation due to the same irradiation dose (8 Gy) is higher for KPC tumor than Pan02 and MC38 tumors, suggesting that KPC cells can be more susceptible to SIRPα-based treatment under RT (Table 2).

For KP1 tumors in NSG mice, we find ICD decreasing with tumor size in combined anti-CD47 and RT treatment (Table 2), consistent with the trend in MC38 tumors in WT mice (Table 1).

Another recent study identified that irradiated colorectal cancer (CRC) cells use the ataxia-telangiectasia and Rad3-related (ATR)-mediated DNA double-strand break repair pathway to upregulate CD47 and PD-L1, which interact with SIRPα and PD-1, respectively, to suppress phagocytosis and tumor-associated antigens (TAA) cross-presentation by APCs (24). RT combined with anti-CD47/anti-SIRPα antibodies enhanced dendritic cell function and CD8 T cell priming and propagated the local tumoricidal activity of RT into vigorous systemic antitumor immunity (24). Our model can fit their MC38-OVA tumor growth data in WT mice with RT and anti-CD47 or anti-SIRPα (Fig. 6*C*) (*SI Appendix*, Table S7). We find that immune activation in the case of anti-CD47 treatment (A=0.27) is lower than in the case of anti-SIRPα treatment (A=0.33), suggesting that anti-SIRPα is more effective than anti-CD47 for MC38-OVA.

Our mathematical model also recapitulates MC38 tumor growth data in WT (C57BL/6) mice with anti-SIRPα and high-dose radiotherapy (HRT) of 12 Gy(25) (Fig. 6*D*). Comparing the ICD activation A values (*SI Appendix*, Table S7), we observe that immunogenic activation with HRT is much lower in WT mice with anti-SIRPα treatment than in SIRPα-deficient mice.

For pancreatic cancer (Pan02 and KPC) growth data in WT and SIRP$\alpha^{-/-}$ mice, we fit $c_{1}$, $c_{\max}$, and A, (Fig. 6 *E* and *F*). We keep the radio-sensitivity parameter the same as MC38 cells to avoid parameter overfitting. Note that RT of 8 Gy is administered on day 12 for Pan02 and day 18 for KPC, respectively, to keep the same tumor sizes of 200 mm^3^. We find A=15.33 for Pan02, comparable to that of MC38 tumors, and A=17.24 for KPC, resulting in higher ICD for KPC than for MC38 and Pan02.

### Model Predicts Abscopal Effect in SIRP*α*^−/−^ Mice.

**Fig. 7.**
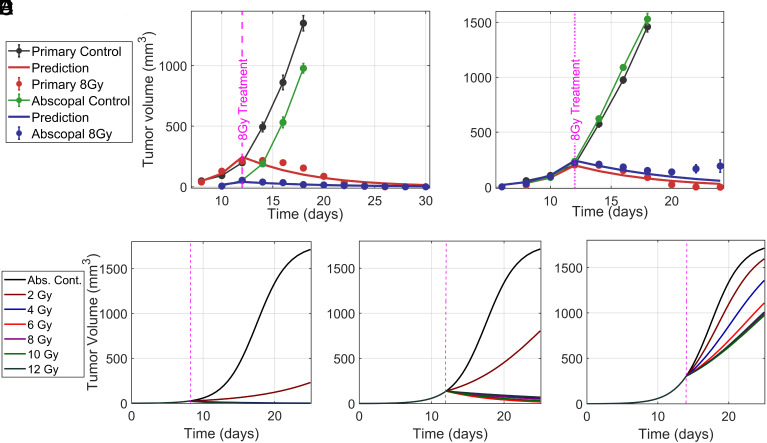
Mathematical model predicts the abscopal effect in MC38 tumors in SIRP$\alpha^{-/-}$ mice: Our model accurately simulates the abscopal effect in MC38 tumors with different initial engraftments, demonstrating the robustness of its prediction for systemic tumor response to localized radiation therapy. (*A*) Initial engraftment of $1\times 10^{5}$ MC38 cells. (*B*) Initial engraftment of $5\times 10^{5}$ cells. (*C*–*E*) Predicts abscopal effect for small (*C*), medium (*D*), and large (*E*) tumors across varying RT doses. A medium dose (≈6 Gy) is most effective for reducing small and medium abscopal tumors, while higher doses yield better outcomes for large tumors. Experimental data in (*A* and *B*) from ref. 22.

The abscopal effect, although rare, is a systemic immune response that reduces tumor burden outside the irradiated area. Our mathematical model successfully predicts the abscopal effect in SIRP$\alpha^{-/-}$ mice and reproduces the tumor growth data reported in ref. 22 without parameter fitting. Interestingly, the model demonstrates a complete reduction of abscopal tumors smaller than <100 mm^3^ (Fig. 7*A*). However, for abscopal tumors larger than 200 mm^3^, the reduction is less pronounced (Fig. 7*B*), with some tumor cells surviving and leading to relapse one week posttreatment. Our model does not effectively capture this relapse, likely due to the simplified assumption of constant ICD values. A more sophisticated model incorporating adaptive immune dynamics may be needed to better describe abscopal tumor relapse.

Additionally, the model predicts abscopal effect across different tumor volumes (Fig. 7*C*–*E*), when the primary tumor is exposed to radiation doses between 2 and 12 Gy. Among these doses, the mid-range dose of ≈6 Gy stands out as most effective in reducing the small (<50 mm^3^, Fig. 7*C*) and medium-sized (50 to 200 mm^3^, Fig. 7*D*) abscopal tumors. For large abscopal tumors (>200 mm^3^, Fig. 7*E*), higher RT doses (>6 Gy) yield better outcomes, mirroring the response observed in primary tumors (Figs. 3 and 7*E*).

### Treatment Efficacy Depends on Radiation Dose and Immunogenic Activation.

We analyze the effects of varying radiation dose (d) for different values of the immune activation (A) and macrophage phagocytosis ($\phi_{m}$) on the efficacy of tumor treatment. Treatment efficacy is defined as the percent reduction in tumor volume C:

$$\begin{array}{c} \text{Treatment Efficacy}=\left(\frac{C_{\text{no treatment}}-C_{\text{treatment}}}{C_{\text{no treatment}}}\right)\times 100 \end{array}$$

**Fig. 8.**
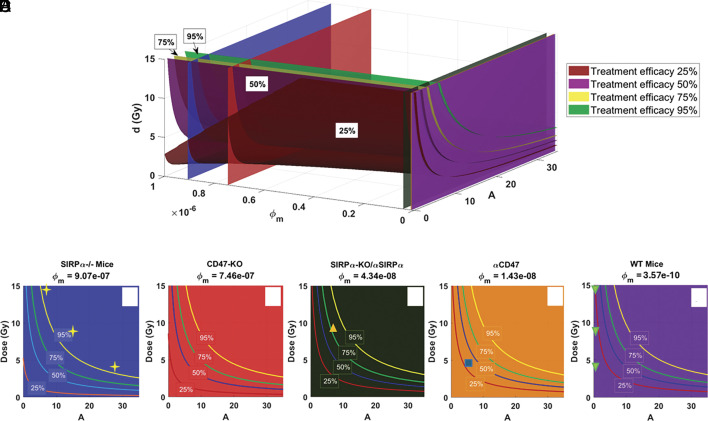
Predicted treatment efficacy for combined RT and macrophage-based immunotherapy: (*A*) Isosurfaces at four efficacy levels (25%, 50%, 75%, and 95%) as a function of ICD activation A, macrophage phagocytosis $\phi_{m}$, and radiation dose. (*B*–*F*) Cross-sections of efficacy contours for $\phi_{m}$ values corresponding to various treatment options for inhibiting the SIRPα-CD47 checkpoint. Yellow stars in (*B*) correspond to data in SIRP$\alpha^{-/-}$ mice with three irradiation doses (data from ref. 22). Yellow upward triangle in (*D*) corresponds to twice i.t./i.v. injections of SIRP$\alpha^{-}$ macrophages into WT mice (data from ref. 22), blue square in (*E*) is for anti-CD47 treatment (data from ref. 23), and green downward triangles in (*F*) are for WT mice (experimental data from ref. 22).

Using the mathematical model, we simulate tumor growth dynamics varying $\phi_{m}$, A, and d values, and illustrate the isosurfaces of 25%, 50%, 75%, and 95% treatment efficacy in a three-dimensional (3D) phase space (Fig. 8*A*). In all these cases, the combined RT and checkpoint inhibition is applied on day 12 (intermediate tumor volume), and treatment efficacy is evaluated on day 22. Day 22 was chosen because it comes after all RT application days in the datasets (e.g., days 5 to 18), allowing fair assessment of treatment effects. It also marks the latest time point with consistent control tumor data across all schedules in the (22) dataset, making it the most suitable and comparable endpoint. We then map the specific treatments of SIRPα-CD47 inhibition for MC38 tumor cells in WT and SIRPα-deficient mice according to their phagocytosis values ($\phi_{m}$) as cross-sections through the 3D space. SIRPα-KO and anti-SIRPα treatments result in similar $\phi_{m}$ values and are therefore grouped together. Sequentially from left to right in the cross-sectional views, we see all the treatment efficacy lines shift upward, suggesting that the irradiation dose required to achieve the same treatment efficacy increases. In other words, for medium-sized tumors (100 to 400 mm^3^), given the same irradiation dose, CD47-KO is more effective in reducing the tumor than SIRPα-KO and anti-SIRPα, which in turn is more effective than anti-CD47. The 3D efficacy map for small (<100 mm^3^) and large (>600 mm^3^) is only slightly different (*SI Appendix*, Fig. S8).

A different way to look at the treatment efficacy is presented as a collection of heatmaps for different doses (*SI Appendix*, Fig. S9). With the model calibrated for MC38 tumors in WT mice, considering both an increased phagocytosis rate $\phi_{m}$ due to SIRPα-CD47 checkpoint inhibition and an irradiation dose-dependent ICD, we evaluate treatment efficacy when RT is initiated on days 8, 12, and 14, corresponding to small (<100 mm^3^), intermediate (100 to 400 mm^3^), and large (400 to 600 mm^3^) tumors, respectively. These heatmaps can serve as a guide for optimal RT doses given the tumor size and treatment options.

## Discussion

Cancer cells evade immune destruction through multiple mechanisms, with inhibition of innate recognition and innate-to-adaptive activation of T cell immunity being a major obstacle in immunotherapy. Immunogenic cell death (ICD) has emerged as a key process that enhances antitumor immunity by triggering specific tumor-associated antigens (e.g., CRT, HSP70/90, ATP, and HMGB) (5) and activating immune cells. Our study presents a mathematical model to quantify the role of ICD in enhancing combined RT and macrophage-based immunotherapy, specifically targeting the SIRPα-CD47 checkpoint. While macrophage-based immunotherapies have shown potentials in preclinical murine models, clinical translation remains challenging. CD47 blockade alone has shown tissue toxicity and limited efficacy in human trials (36–38). SIRPα blockade also exhibits unintended phagocytosis of healthy CD47-expressing cells (39, 40), while combination therapies targeting the SIRPα-CD47 checkpoint show promise (37, 41–43). These findings underscore the need for optimized combination therapies to enhance efficacy while minimizing safety risks.

Our model, developed through a robust 4-step process, provides several insights into the interplay between RT, immune activation, and phagocytosis activation. We find that RT alone fails to generate a sufficient ICD response in WT mice, even at higher doses, highlighting a key limitation of RT-based immunotherapy: Without additional immune activation, RT-induced tumor cell death remains largely nonimmunogenic. Our model predicts that disrupting the SIRPα-CD47 checkpoint enhances macrophage-mediated phagocytosis, shifting the tumor microenvironment toward a more immune-active state.

Our results identify an optimal radiation dose range (6 to 8 Gy) that maximizes ICD induction without excessive immune suppression. This aligns with previous experimental findings suggesting that intermediate RT doses enhance tumor immunogenicity, whereas higher doses may impair immune activation (31, 32). These findings could provide a quantitative basis for designing clinical trials that optimize radiation dose and scheduling to synergize with macrophage-based immunotherapy.

Several extensions of our model could make it more biologically realistic. For example, prior studies suggest that CD47 expression can be upregulated or downregulated following treatment (35). To capture this CD47 modulation, we can include a treatment-dependent phagocytosis rate. However, detailed quantitative time-course measurements of CD47 expression pre- and posttreatment would be necessary to parameterize and validate such a dynamic extension. Another important avenue is modeling tumor-intrinsic RT resistance. Radio-resistance can be incorporated by modifying the radiosensitivity parameters (α,β) in the linear-quadratic model. Lower α and β values correspond to higher survival fraction and lower tumor damage, which diminishes ICD induction, and ultimately significantly reduces treatment efficacy. Our preliminary simulations (details in *SI Appendix*) suggest that even a small RT-resistance results in a significant reduction in treatment efficacy, especially for larger tumors and at lower doses. The impact is dose and tumor size-dependent. In larger tumors, optimal RT dose may not exist, raising important questions about dose escalation vs. combinatorial treatment strategies.

We predict that local RT combined with macrophage activation can induce a systemic immune response, reducing tumor burden beyond the irradiated site (abscopal effect). This result highlights that immune checkpoint inhibitors targeting SIRPα-CD47 amplify the systemic impact of radiotherapy, providing a rationale for combination strategies in metastatic cancer treatment. In these combined RT and SIRPα-CD47 checkpoint inhibition treatments, through quantification of enhanced immunogenic activities and ICD, we demonstrate that the primary role of macrophages is the induction of a systemic immune response and the promotion of T cell cytotoxicity that produces additional ICD, not direct tumor killing. Notably, this framework provides a predictive tool for optimizing combination therapies, refining radiation dosing, and quantifying systemic immune responses.

Despite these advances, several challenges remain. While our model captures key aspects of tumor–immune dynamics, further refinements incorporating adaptive immune responses (e.g., T cell priming and antigen presentation dynamics) would enhance predictive power. Additionally, translating these findings to human tumors requires further validation, as tumor heterogeneity and immune landscape variations may influence ICD induction and treatment efficacy.

Our model parameters align with those reported in tumor dynamics studies. In addition to quantifying the effects of radiation and immunotherapy, our model critically evaluates the role of macrophage type in tumor dynamics. Unlike mathematical models that classify macrophages into distinct M1 and M2 subtypes, our approach accounts for the dynamically altered phagocytosis rates resulting from the perturbations in the CD47-SIRPα checkpoint. This perspective aligns with recent findings that view macrophage phenotypes as a continuous and context-dependent spectrum rather than a rigid, bipolar M1/M2 classification (44).

To improve the translational relevance of our findings, future studies can focus on experimental validations in human tumor models by expanding preclinical datasets to patient-derived xenografts and organoid models, and assessing how tumor-intrinsic factors affect ICD response. Another important area of future model development is to incorporate adaptive immunity, particularly immune memory formation, to study longer-term cancer immunity as reported in refs. 22 and 24. In addition, we can investigate how ICD and macrophage activation interact with checkpoint inhibitors targeting the PD-1/PD-L1 axis.

This study provides a quantitative framework for understanding and optimizing ICD-driven cancer therapy. By demonstrating that SIRPα-CD47 checkpoint inhibition enhances RT-induced ICD, we highlight a potential strategy for improving combination immunotherapies. These findings lay the groundwork for rational treatment design and provide insights into the systemic immune effects of radiotherapy.

## Methods

## Materials and Methods

### Mathematical Model of In Vivo Tumor Growth with Combination Therapy.

The model construction follows a four-step process. 1. Baseline tumor growth in WT mice: Develop a model for in vivo tumor growth in wild type mice, calibrating parameters using the experimental data. 2. Tumor growth in SIRP$\alpha^{-/-}$ mice: Extend the baseline model by incorporating SIRP$\alpha^{-}$ macrophages to simulate tumor growth in knockout mice. 3. Radiotherapy in WT mice: Integrate radiation-induced tumor cell death into the baseline WT tumor model. 4. Radiotherapy in SIRP$\alpha^{-/-}$ mice: Combine the models from steps 2 and 3, incorporating ICD activated by SIRP$\alpha^{-}$ macrophages.

The model assumes that radiation-damaged tumor cells release DAMPs, which activate SIRPα-deficient macrophages, triggering a systemic proinflammatory response that enhances tumor clearance.

#### Step 1: Tumor growth in WT mice.

Various models can describe in vivo tumor growth, ranging from simple logistic growth models to complex immune interaction models. We choose the simplest model that best fit the data and captures the biological dynamics (details in *SI Appendix*).

The cancer–immune interaction model, termed the CE model, is

[1] $$\begin{array}{c} \text{Cancer cell:}\;\frac{\mathrm{dC}}{\mathrm{dt}}=c_{1}C\left(1-\frac{C}{c_{\max}}\right)-\phi_{e}CE. \end{array}$$

[2] $$\begin{array}{c} \text{Effector cell:}\;\frac{\mathrm{dE}}{\mathrm{dt}}=\gamma_{e}-\eta_{e}CE-\delta_{e}E. \end{array}$$

The cancer cell population C grows logistically at a rate $c_{1}$ with carrying capacity $c_{\max}$, while $\phi_{e}$ represents cancer cell clearance by effector cells E. The tumor-associated effector cells E infiltrate the tumor at a rate $\gamma_{e}$ and are cleared at an intrinsic rate of $\delta_{e}$ and an exhaustion rate $\eta_{e}$ from combating the tumor cells.

Tumor and effector cell parameters are fitted using WT tumor growth data and kept constant in subsequent steps.

#### Step 2: Modeling tumor growth in SIRP$\alpha^{-/-}$ and CD47-SIRPα treated mice.

To model tumor dynamics in SIRP$\alpha^{-/-}$ or CD47/SIRPα-targeted treatments, we introduce a new cell population $M^{*}$ for treated macrophages, which exhibit enhanced phagocytosis. The cancer-effector-macrophage (CEM) model is

[3] $$\begin{array}{c} \text{Cancer cell:}\;\frac{\mathrm{dC}}{\mathrm{dt}}=c_{1}C\left(1-\frac{C}{c_{\max}}\right)-\phi_{e}CE-\phi_{m}^{*}CM^{*}. \end{array}$$

[4] $$\begin{array}{c} {\text{Macrophage}}^{*}\text{:}\;\frac{dM^{*}}{\mathrm{dt}}=\gamma_{m}^{*}-\eta_{m}^{*}CM^{*}-\delta_{m}^{*}M^{*}, \end{array}$$

where $\phi_{m}^{*}$ is phagocytosis rate. Similar to the effector cells, macrophages with CD47-SIRPα treatment have a tumor infiltration rate $\gamma_{m}^{*}$, an exhaustion rate $\eta_{m}^{*}$, and a natural decay rate $\delta_{m}^{*}$. The effector cell dynamics remain the same as Eq. **2**.

Four additional parameters related to SIRPα-deficient macrophage dynamics were fitted using SIRP$\alpha^{-/-}$ and CD47-SIRPα treated tumor growth data.

#### Step 3: RT in WT mice.

The impact of RT administered at $t_{1}$ is modeled by modifying the tumor cell growth as

[5] $$\begin{array}{c} {\left(\frac{\mathrm{dC}}{\mathrm{dt}}\right)}^{+}=S{\left(\frac{\mathrm{dC}}{\mathrm{dt}}\right)}^{-}. \end{array}$$

The survival fraction S follows the linear-quadratic (LQ) model as a function of radiation dose d (32):

[6] $$\begin{array}{c} S=e^{-\alpha d-\beta d^{2}g\left(\lambda \tau \right)}, \end{array}$$

where α and β are the susceptibility to direct DNA damage and complex damage dynamics involving repair processes, respectively, and $g\left(\lambda \tau \right)=2\left(\lambda \tau +e^{-\lambda \tau }-1\right)/{\left(\lambda \tau \right)}^{2}$ is the repair function, τ is the RT delivery time (an experimental constant), and λ is the repair parameter (32). LQ model parameters are fitted using WT tumor growth data under RT.

#### Step 4: RT in SIRP$\alpha^{-/-}$ mice or CD47/SIRPα treated mice.

In SIRP$\alpha^{-/-}$ mice or CD47/SIRPα-treated mice, increased tumor cell death is attributed to ICD, modeled as

[7] $$\begin{array}{c} {\left(\frac{\mathrm{dC}}{\mathrm{dt}}\right)}^{+}=S{\left(\frac{\mathrm{dC}}{\mathrm{dt}}\right)}^{-}-I{\left(\frac{\mathrm{dC}}{\mathrm{dt}}\right)}^{-}, \end{array}$$

where I=A(1−S) represents immunogenic cell death and A is the immune activation rate. All the other equations and their parameters remain the same as in Steps 2 and 3. The only fitting parameter is the immune activation parameter A for different RT doses and tumor volumes.

#### Abscopal tumor growth in SIRP$\alpha^{-/-}$ or CD47/SIRPα treated mice with RT.

The nonirradiated (out-of-field or abscopal) tumor shares intrinsic growth kinetics and immune context with the primary tumor because they arise from the same cell line within the same host. The key difference lies in the absence of a direct radiation dose to the out-of-field tumor. Its dynamics is described as

[8] $$\begin{array}{c} {\left(\frac{\mathrm{dC}}{\mathrm{dt}}\right)}^{+}={\left(\frac{\mathrm{dC}}{\mathrm{dt}}\right)}^{-}-I{\left(\frac{\mathrm{dC}}{\mathrm{dt}}\right)}^{-}, \end{array}$$

without S and with the same I as in the primary tumor (Eq. **7**). All the other equations and parameters remain the same as in the Steps above.

Detailed descriptions of model development and parameter estimation are in *SI Appendix*. All parameters fall within ranges reported in the literature (*SI Appendix*, Table S7).

### Parameters Uncertainty Quantification.

To assess parameter uncertainty, we employ a parametric bootstrapping strategy, assuming that the time-series data follow a Poisson distribution centered on model predictions at each time point (45). We generate bootstrap samples by randomly sampling n=2,000 times (with replacement) from the original data points. The model is then fitted to the bootstrapped datasets using the least squares method, yielding 2,000 sets of parameter estimates. From this distribution, we compute the mean and associated uncertainty for each parameter.

### Parameter Sensitivity Analysis.

We perform a local sensitivity analysis to assess how small variations in each parameter affect the model’s output. Parameter ranges are determined through uncertainty quantification, and their mean values are the best-fit estimates. To assess sensitivity, each parameter is independently perturbed by 25% of its best-fit value, and the resulting relative change in the cost function is measured. The average sensitivity of each parameter is computed across all data points, which forms the basis for a sensitivity ranking that identifies the most and least influential parameters.

### Parameter Identifiability Analysis.

We assess both structural and practical identifiability of the model parameters. Structural identifiability is evaluated using the STRIKE-GOLDD toolbox in MATLAB and the StructuralIdentifiability.jl package in Julia. Practical identifiability is examined through profile likelihood analysis and by quantifying parameter covariability based on the available in vivo tumor growth data. Importantly, even when individual parameters are found to be practically unidentifiable, certain parameter combinations remain identifiable. Further details and full results are provided in *SI Appendix*.

## Supplementary Material

Appendix 01 (PDF)

## Data Availability

Simulated data and code data have been deposited in GitHub (https://github.com/yijianglanl/SIRPa-Paper-Code) Yi Jiang. yijianglanl/SIRPa-Paper-Code: SIRPa-Paper-Code v1.0.0. Zenodo; 2025 (46). All other data are included in the manuscript and/or *SI Appendix*. Previously published data were used for this work (22–25).
