# Supplementary material for: Immunogenic cell death unlocks the potential for combined radiation and immunotherapy
Source: Proc Natl Acad Sci U S A. 2025 Nov 26;122(48):e2509875122. doi: 10.1073/pnas.2509875122 (PMC12685091; doi:10.1073/pnas.2509875122)
Supplement: Supplementary file 1 — Appendix 01 (PDF) [file pnas.2509875122.sapp.pdf]

# Supporting Information for

## Immunogenic Cell Death Unlocks the Potential for Combined Radiation and Immunotherapy

**Somiya Rauf, Alexandra Smirnova, Andres Chang, Yuan Liu, Yi Jiang**

**Corresponding Author: Yi Jiang, Email: yjiang12@gsu.edu**

### This PDF file includes:

Supporting text
Figs. S1 to S9
Tables S1 to S7
SI References

## Supporting Information Text

## Model development

*"Everything should be made as simple as possible, but not simpler."* – Albert Einstein

**Tumor growth in WT mouse: evidence of limited macrophage phagocytosis.** We strive to develop the simplest mathematical model that can explain the complex dynamics of *in vivo* tumor growth with immunotherapy and radiotherapy (RT), given the extensive availability of *in vivo* tumor growth data with combination immuno- and radio-therapy (1–4). In our step-by-step model development, we start with a basic ODE model that captures the dynamic interactions between immune and tumor cells, adhering to the methodologies and principles established in prior studies (5–7). Our first step is to accurately describe *in vivo* tumor growth observed in WT mice, with the influence of immune cells within the tumor microenvironment (TME). When we consider the WT macrophages $M$ as well as the effector cells $E$, we have

$$\begin{aligned} &\text{Cancer:} && \frac{dC}{dt} = c_1 C\left(1 - \frac{C}{c_{\max}}\right) - \phi_e CE - \phi_m CM, \\ &\text{Effector:} && \frac{dE}{dt} = \gamma_e - \eta_e CE - \delta_e E, \\ &\text{WT Macrophage:} && \frac{dM}{dt} = \gamma_m - \eta_m CM - \delta_m M. \end{aligned} \qquad [S1]$$

The cancer cell population follows logistic growth with a rate $c_1$ and a carrying capacity $c_{\max}$, while tumor clearance is mediated by effector cells $E$ and WT macrophages $M$, with respective killing rates $\phi_e$ and $\phi_m$. Effector cells and WT macrophages have similar dynamics, characterized by tumor infiltration rates $(\gamma_e, \gamma_m)$, exhaustion rates $(\eta_e, \eta_m)$, and natural clearance rates $(\delta_e, \delta_m)$. Given the complexity of the TME and immune system, our decision to focus on $C$, $E$, and $M$ is deliberate for both biological and mathematical reasons. Tumor cells are the primary target population driving disease progression. Effector cells represent the main adaptive cytotoxic response, while macrophages capture the innate immune component that is central to the SIRP$\alpha$-CD47 checkpoint under study. These three populations represent the dominant drivers of the phenomena we aim to model: phagocytosis, ICD, and tumor–immune interplay under radiotherapy. Other immune players (e.g., dendritic cells, NK cells, and regulatory T cells) are implicitly represented through effective terms: dendritic cell priming is incorporated into the effector cell activation rate, NK-like killing is approximated within effector cytotoxic activity, and immune suppression is reflected in exhaustion and clearance terms.

From a mathematical perspective, it is impossible and unnecessary to include every aspect of every process involved in tumor growth *in vivo* and the changing TME under RT and immunotherapy. Parsimony is critical to ensure interpretability, identifiability, and reliable calibration with the available *in vivo* tumor growth data. Introducing additional cell populations without sufficient quantitative measurements would lead to parameter unidentifiability and reduce predictive power. As we will show through the step-by-step model development and calibration, our simple three-population model strikes a balance between biological realism and analytical tractability, allowing us to rigorously quantify ICD and macrophage checkpoint modulation while still capturing essential treatment effects. We note that the framework is readily extensible: additional immune populations or cytokine signaling modules could be incorporated as more detailed quantitative data become available. For the present study, however, focusing on these three key populations provides a minimal yet sufficient system to test our hypotheses and generate robust predictions.

In the experiment, varying numbers of MC38 cells ($5 \times 10^3$, $5 \times 10^4$, $5 \times 10^5$, or $2 \times 10^6$) were injected subcutaneously (s.c.) into WT and SIRP$\alpha^{-/-}$ mice (1). The majority of the injected tumor cells die within 24-48 hours due to the host immune response and the challenging new microenvironment, leaving only a subset that survives and successfully establishes tumor growth (8). The initial number of engrafted cancer cells after s.c. injection is critical for subsequent tumor growth. To accurately represent the growth dynamics across these four tumor injections, in addition to the parameters in equation set S1, we also fit for the initial tumor size and the initial effector cell and macrophage populations in the tumor. Tumor volume comprises cancer cells, effector cells, and macrophages.

We begin parameter estimation by generating 2000 bootstrap samples through resampling the original data with replacement. For each bootstrapped dataset, the model was fit using the least-squares method (`lsqcurvefit` in MATLAB), where the cost function is defined as the sum of squared errors. This procedure produces 2000 sets of parameter estimates. For each parameter, the mean and the 95% confidence intervals are derived from the distribution (Supplementary Table S1). The model's goodness-of-fit is quantified by the error rate $||Y_{fit} - Y_{data}||/Y_{data}$.

This three-population CEM model (Supplementary Figure S1A), which includes WT macrophages, achieves a fit comparable to the simpler two-population CE model that only includes cancer and effector cells, with error rates of 5.2% and 4.3%, respectively. However, the fitted macrophage killing rate is an order of magnitude lower than reported values for macrophage phagocytosis, while the macrophage clearance rate is 10-fold smaller (Supplementary Table S1), suggesting that WT macrophages exhibit limited phagocytotic capability in the observed tumor growth data. Moreover, the low sensitivity of macrophage-related parameters (Supplementary Figure S1B) suggests that they contribute minimally to model dynamics. It aligns with the understanding that tumor-associated macrophages (TAMs), despite their volumetric significance, are more M2-like and do not directly engage in cytotoxic activities within the TME (9). Based on macrophage-limited activity to kill tumor cells, we simplify the *in vivo* tumor growth model by excluding WT macrophage-mediated phagocytosis, reducing model complexity without compromising accuracy.

**Evaluation of tumor growth models.** As many mathematical models can describe tumor growth dynamics, we further examine if the logistic growth model provides the best fit to the data. These models are evaluated for their ability to fit the *in vivo* tumor growth data (Supplementary Table S2). The Gompertz model exhibits the highest error rate (41.4%), indicating its limitations in this context. The Logistic, Blumberg's, Richards', and Generalized Logistic provide moderate fits with comparatively low error rates. The exponential model shows a relatively higher error rate (11.6%), suggesting that its simplicity may not adequately capture the complex tumor growth dynamics. Models incorporating strong and weak Allee effects offer additional insights into tumor-immune interactions, emphasizing the role of effector cell density in effective tumor suppression. Ultimately, the logistic growth model is chosen, as it achieves an optimal trade-off between complexity and accuracy while minimizing the risk of overfitting.

$$\begin{aligned} \frac{dC}{dt} &= c_1 C\left(1-\frac{C}{c_{\max}}\right) - \phi_e CE, \\ \frac{dE}{dt} &= \gamma_e - \eta_e CE - \delta_e E. \end{aligned} \quad \text{[S2]}$$

**Tumor growth in SIRP$\alpha^{-/-}$ mice: enhanced phagocytosis for SIRP$\alpha^-$ macrophages.** To model tumor growth in SIRP$\alpha^{-/-}$ mice, we introduce SIRP$\alpha$-deficient macrophages, denoted as $M^*$. These SIRP$\alpha$-deficient macrophages show enhanced tumor cell phagocytosis, table S4. The equation for $M^*$ is similar to that of WT macrophage, incorporating a tumor infiltration rate, an exhaustion rate, and a baseline clearance rate:

$$\begin{aligned} \text{Cancer:} \quad & \frac{dC}{dt} = c_1 C\left(1-\frac{C}{c_{\max}}\right) - \phi_e CE - \phi_m^* CM^*, \\ \text{Effector:} \quad & \frac{dE}{dt} = \gamma_e - \eta_e CE - \delta_e E, \\ \text{SIRP}\alpha^{-/-} \text{ Macrophage:} \quad & \frac{dM^*}{dt} = \gamma_m^* - \eta_m^* CM^* - \delta_m^* M^*. \end{aligned} \quad \text{[S3]}$$

To fit the parameters for tumor growth in SIRP$\alpha^{-/-}$ mice, we keep the six parameters associated with the tumor cells and effector cells unchanged and focus solely on fitting the four parameters related to macrophages using the same uncertainty quantification analysis. We find that SIRP$\alpha^-$ macrophages exhibit superior phagocytic activity (Supplementary Table S4), two orders of magnitude higher than the reported value for M1 macrophages (10). This observation agrees with experimental findings (1, 11) which report the enhanced phagocytic capability of SIRP$\alpha^-$ macrophages and SIRP$\alpha$-treated macrophages. This heightened phagocytic capability contributes to the observed deceleration in tumor growth when exposed to a lower inoculum, highlighting the enhanced immune control exhibited by SIRP$\alpha^{-/-}$ mice *in vivo*.

**Modeling Radiotherapy (RT).** For RT applied to tumors in WT mice, we start with the WT tumor growth model S2 and introduce RT as a stepwise modification to the continuous equation. Instead of assuming instantaneous tumor cell elimination upon application, we account for the prolonged effects of RT, as its impact extends over time (12). To avoid introducing excessive parameters, we do not explicitly model CD47 induction under RT; therefore, we keep the phagocytosis rate the same as in the absence of RT treatment for mono RT treatments.

To investigate tumor response across various radiation doses, we incorporate RT-induced cancer cell death using a linear-quadratic dose-dependent survival fraction (13, 14).

$$S = e^{-\alpha d - \beta d^2 g(\lambda\tau)}, \quad \text{[S4]}$$

where $\alpha$ and $\beta$ are the susceptibility to direct DNA damage and complex damage dynamics involving repair processes, respectively, and $g(\lambda\tau)$ is the repair function:

$$g(\lambda\tau) = 2\frac{\lambda\tau + e^{-\lambda\tau} - 1}{(\lambda\tau)^2}, \quad \text{[S5]}$$

where $\tau$ is the duration of radiation delivery and $\lambda$ is the repair parameter (13). Reported radiation delivery rates vary across studies, such as 1.2 Gy/min in (1), 2.41 Gy/min in (2), 2.53 Gy/min for (15). We calculate $\tau$ for each dose in each experiment accordingly.

Irradiation activates the immune system, prompting the release of cytokines, chemokines, and growth factors, which play crucial roles in regulating immune responses, inflammation, cell recruitment, tissue growth, and repair (16). Assuming the amount of DAMPs generated by radiation is proportional to the fraction of damaged cancer cells $(1-S)C$ and immunogenic cell death $I$ is proportional to damaged cancer cells $(1-S)C$, with a proportionality constant $A$. This proportionality constant depends on RT dose, tumor size, and TME. The cancer cell population post-RT grows at a fraction $S$ of its pre-RT rate and decays at a rate proportional to $1-S$:

$$\left(\frac{dC}{dt}\right)^+ = S\left(\frac{dC}{dt}\right)^- - A(1-S)\left(\frac{dC}{dt}\right)^-. \quad \text{[S6]}$$

Supplementary Table S5 summarizes the parameters in the linear-quadratic model. The calibrated parameter values show minimal $A$ and $I$ values, suggesting limited ICD in WT mice after RT. We combine the model for WT tumor growth model (Equation S2) and for the SIRP$\alpha^{-/-}$ tumor growth model (Equation S3) and RT (Equations S4, S5 and S6) to model RT in WT and SIRP$\alpha^{-/-}$ mice, respectively. A significantly enhanced ICD is observed for each dose and varying tumor volumes.

**Modeling Radiotherapy (RT) Resistance.** In our model, radiotherapy exerts three effects: (i) direct tumor cell killing, (ii) immune activation via ICD, and (iii) collateral damage to immune cells. Radiation resistance can be incorporated into the model as a modification of RT parameters, e.g., smaller $\alpha$, $\beta$ values in the linear-quadratic formula, corresponding to increased survival fraction after RT and ICD reduction. ICD-related effects of RT (immune activation versus immune damage) may exhibit different sensitivities to dose, and resistance would primarily diminish the tumor-directed damage due to RT resistance. To illustrate this explicitly, we can modify our RT application equation to include a resistance term:

$$\left(\frac{dC}{dt}\right)^+ = (S_1+\epsilon)\left(\frac{dC}{dt}\right)^- - A(1-(S_1+\epsilon))\left(\frac{dC}{dt}\right)^-, \qquad \text{[S7]}$$

where $S = S_1 + \epsilon$ the increased survival fraction due to RT resistance, $\epsilon$ corresponds to the degree of RT resistance. We illustrate the effects of RT resistance $\epsilon = 0.05$ using MC38 tumor growth in SIRP$\alpha$-deficient mice (1). Using all calibrated parameters, we compare the growth curves with and without resistance for small (RT on day 8), medium (day 12), and large (day 14) tumors at two doses, 4 and 8 Gy, respectively. Figure S2 shows that the effect of RT resistance depends on tumor size and RT dose: Only for small tumors at large doses is the impact of resistance minimal; for smaller doses or larger tumors, the impact of small resistance is drastic; for large tumors, the treatment efficacy is diminished.

## Sensitivity analysis

We use a systematic perturbation approach to assess the sensitivity of parameters.

**Local Sensitivity.** The perturbation factor, denoted by $f$, is set to $f = 0.25$. $X_p$ the perturbed parameter matrix with $x_p(x) = x \pm \epsilon(x)$ the perturbed value. The sensitivity matrix is $S(p) = \frac{Y(x_p(x)) - Y_{fit}}{Y_{fit}}$, where $Y_{fit}$ is the model fit obtained from the mean parameter value $(x)$ computed in uncertainty quantification. The sensitivity index for each parameter is computed as the average of the absolute values in the sensitivity matrix. This index quantifies the relative importance of each parameter in influencing the model predictions. In the WT CEM and CE models, we find that the tumor cell growth rate, $c_1$, and the maximum tumor capacity, $c_{\max}$, are the most sensitive parameters, affecting tumor growth dynamics significantly, followed by the parameters related to the effective cells $\phi_e$ and $\gamma_e$, while the effector cell exhaustion rate is less sensitive (Supplementary Figures S1B and S3A). Parameters associated with macrophages in the CEM model show minimal influence on the model's behavior (Supplementary Figure S1B). On the other hand, in the SIRP$\alpha^{-/-}$ mice, $\phi_m^*$ and $\eta_m^*$ emerge as sensitive parameters, underscoring the key role of SIRP$\alpha^-$ macrophages in the tumor dynamics (Supplementary Figure S3B). Moreover, when RT is applied, the sensitivity analysis shows the time-dependent impact of RT parameters $\alpha, \beta$, and $\lambda$. In WT mice, these parameters exert minimal influence with sensitivity values less than $10^{-3}$ (Supplementary Figure S4A). Conversely, in SIRP$\alpha^{-/-}$ mice, the relative sensitivity RT parameters are higher (Supplementary Figure S4B), highlighting the altered dynamics due to the immune profile modification.

**Global Sensitivity.** Sobol sensitivity analysis provides the variance of the model output into contributions from all parameters separately. The first-order Sobol sensitivity index for a parameter $x_j$ is defined as:

$$S_1^j = \frac{\mathrm{Var}_{x_j}\left(E_{x_{\sim j}}[Y \mid x_j]\right)}{\mathrm{Var}(Y)},$$

where $S_1^j$ reflects the direct effect of the parameter on the output. Var is the variance, and the term $E_{x_{\sim j}}[Y \mid x_j]$ is the expected value of $Y$ given $x_j$, averaged over all other parameters (17). Supplementary Figure S5 shows the global sensitivity of all parameters for the SIRP$\alpha^{-/-}$ mice case when RT is applied to a small tumor. The tumor dynamics parameters $c_1$ and $c_{max}$ and RT parameters appear to be highly influential, consistent with our findings from the local sensitivity analysis (Supplementary Figure S3).

## Parameters Identifiability

**Structural Identifiability.** We assess parameter identifiability using two independent tools: STRIKE-GOLDD (MATLAB toolbox v4.2.0) and the StructuralIdentifiability.jl package (Julia). Both methods confirm that the parameters in our models for WT tumor growth and SIRP$\alpha^-$ tumor growth, with and without radiotherapy, are structurally identifiable, both locally and globally, from the available measurements. Specifically, the models are identifiable from observed dynamics of total cell populations ($C + E$ for WT and $C + E + M^*$ for SIRP$\alpha^-$), indicating that the proposed formulations are theoretically sound for parameter estimation.

**Practical Identifiability via Profile Likelihood.** To evaluate the practical identifiability of model parameters, we employed the profile log-likelihood method as implemented in (18), fitting all four experimental datasets simultaneously for all models. Fitting multiple datasets together improves robustness but also permits parameter compensation: a suboptimal parameter value for one dataset may still yield an acceptable global fit due to trade-offs with others. As a result, the goal is not to pinpoint a single "true" value for each parameter but to define confidence intervals over which each parameter remains consistent with the data.

To generate noisy data, we add heteroscedastic noise to the simulated model outputs using the following form:

$$\sigma_i^2 \;=\; (CV \cdot N^{(k)}(t_{k,i}; X, IC))^2 \;+\; \sigma_{\text{floor}}^2,$$

and generate noisy, observed data as follows:

$$N_{obs}^{(k)}(t_{k,i}) \;=\; N^{(k)}(t_{k,i}; X, IC) \;+\; \epsilon, \quad \epsilon \sim \mathcal{N}(0, \sigma^2),$$

where $\boldsymbol{X}$ denotes the model parameters, $IC$ the initial conditions for each dataset $k = 1, ..., 4$, and $N^{(k)}(t; X, IC)$ the model-predicted total cell count. The joint log-likelihood is given by:

$$\ell(X, IC \mid N_{obs}) \;=\; -\frac{1}{2\sigma^2} \sum_{k=1}^{4} \sum_{i \in \mathcal{I}_k} \Big( N_{obs}^{(k)}(t_{k,i}) - N^{(k)}(t_{k,i}; X, IC) \Big)^2 \;-\; \frac{n}{2} \log\big(2\pi\sigma^2\big),$$

where $\mathcal{I}_k$ indexes the observation times for dataset $k$, and $n$ is the total number of observations.

We compute univariate profile log-likelihoods for each of the parameter $x_j$, by optimizing all other parameters at each fixed fixing $x_j$ at

$$\ell_p(x_j) \;=\; \max_{X, IC:\, X_j = x_j} \; \ell(X, IC \mid N_{obs}).$$

The total cell population $N(t)$ corresponds to $C + E$ for the CE model and $C + E + M^*$ for the CEM model. Starting with our WT tumor growth model, for all four datasets to fit together, the profile likelihood curves (Figure S6A) show that parameters $c_1$, $c_{max}$ and $\phi_e$ are practically identifiable while $\gamma_e$, $\eta_e$ and $\delta_e$ are unidentifiable. Bivariate profiles (Figure S7) show compensatory relationships between $\gamma_e$ and $\delta_e$, and negative correlation between $\phi_e$ and $\eta_e$. In the SIRP$\alpha^{-/-}$ tumor growth model, all fitted parameters are practically non-identifiable (Figure S6B). For WT tumor growth under RT, parameter $\lambda$ is practically identifiable, but $\alpha$ and $\beta$ from the linear-quadratic model are not with only their upper limit bounded. However, the combined survival fraction $S$ is identifiable for all tested RT doses (Figure S6C). Lastly, for SIRP$\alpha^{-/-}$ under RT, the RT-induced ICD scaling factor $A$ is unidentifiable and unbounded above. The ICD value $I = A(1 - S)$ remains unidentifiable. However, combining $I$ and $S$ into $IR = I - S$, we find that $IR_4$ (dose=4 Gy) and $IR_8$ (8 Gy) are practically identifiable but $IR_{15}$ remain unbounded above (Figure S6D). Worth noting that $IR_{15}$ corresponds to the high-dose RT scenario (15 Gy), where tumor volumes ($100 - 400mm^3$) are fully ablated, eliminating identifiability information.

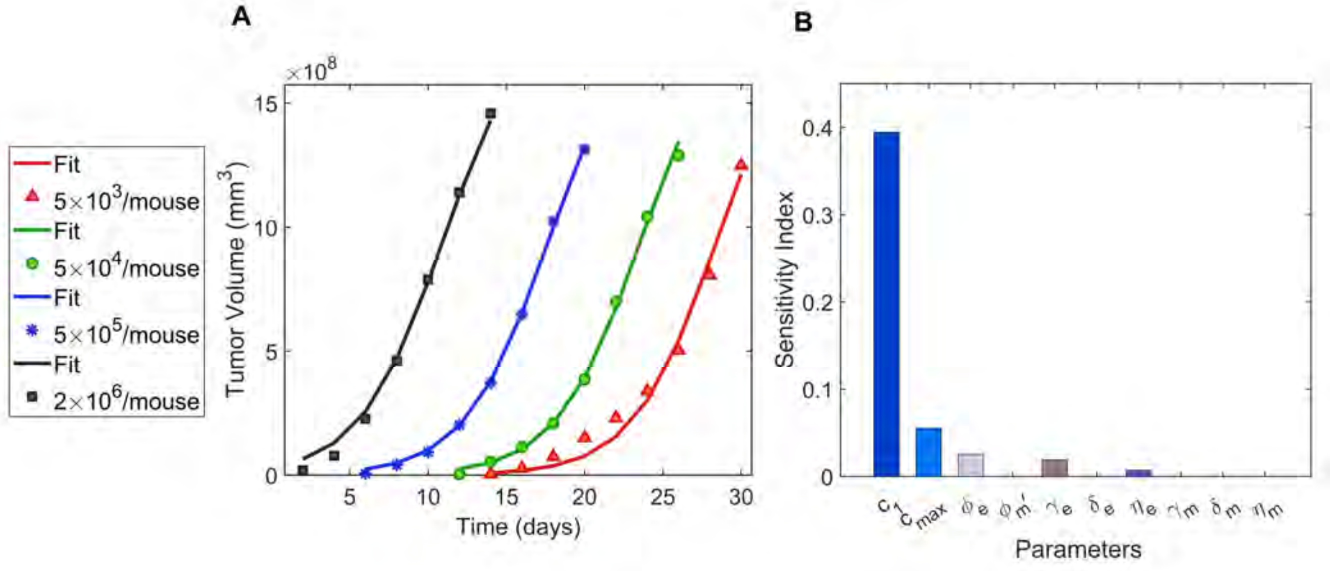


**Fig. S1.** **A.** Model fitting of the CEM tumor growth dynamics to experimental data from WT mice, with colors indicating different subcutaneous (s.c.) MC38 injection numbers. Markers represent experimental data, and solid lines show model fits. **B.** Sensitivity analysis indicates minimal influence of macrophage-specific parameters, suggesting they may be non-essential in WT mice.

 

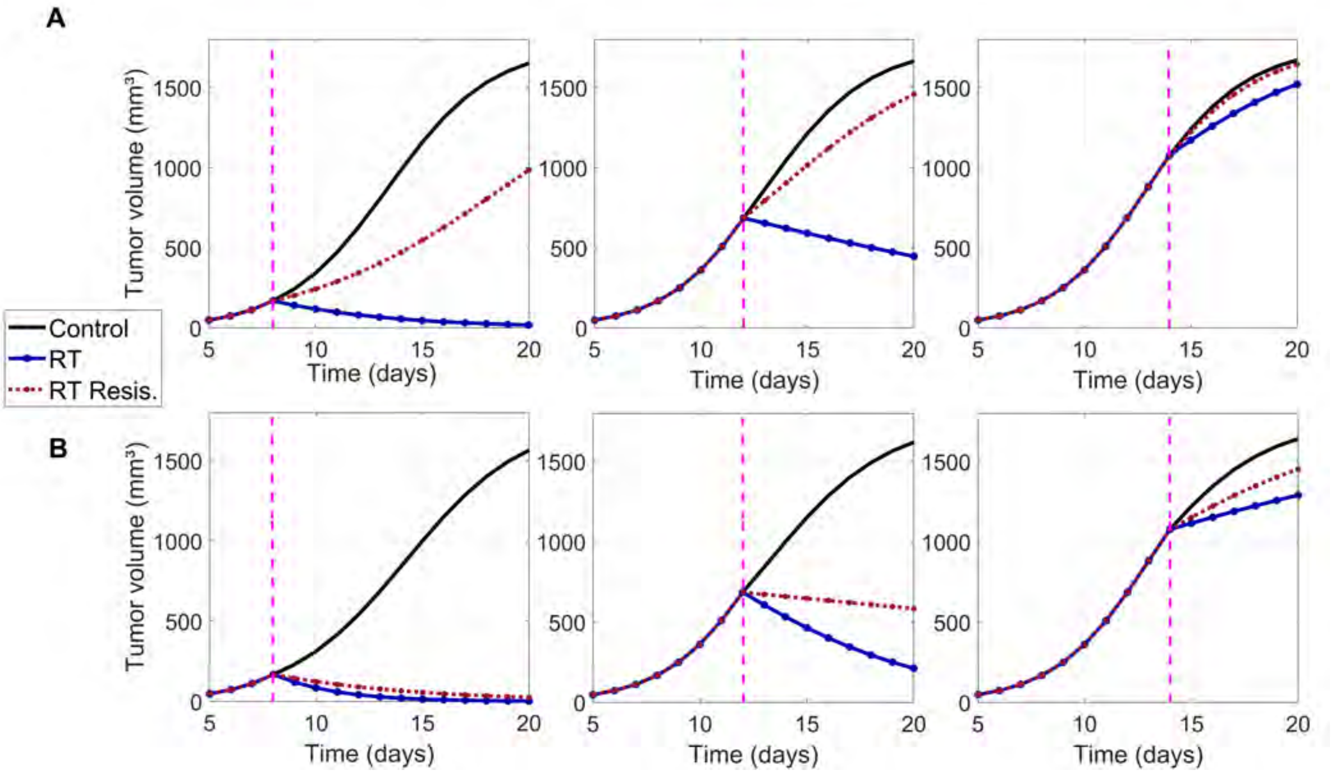


**Fig. S2.** Impact of RT resistance on MC38 tumor growth in SIRP$\alpha^{-/-}$ mice. **A.** Tumor growth under 4 Gy RT administered on day 8, 12, and 14 (left to right). **B.** Tumor growth under 8 Gy RT administered on the same respective days. Black solid lines are untreated control, blue solid lines with dots are RT-treated tumors with normal sensitivity, and red dashed lines are RT-treated tumors with resistance ($\epsilon = 0.05$). The vertical magenta dashed lines mark the time of radiation delivery.

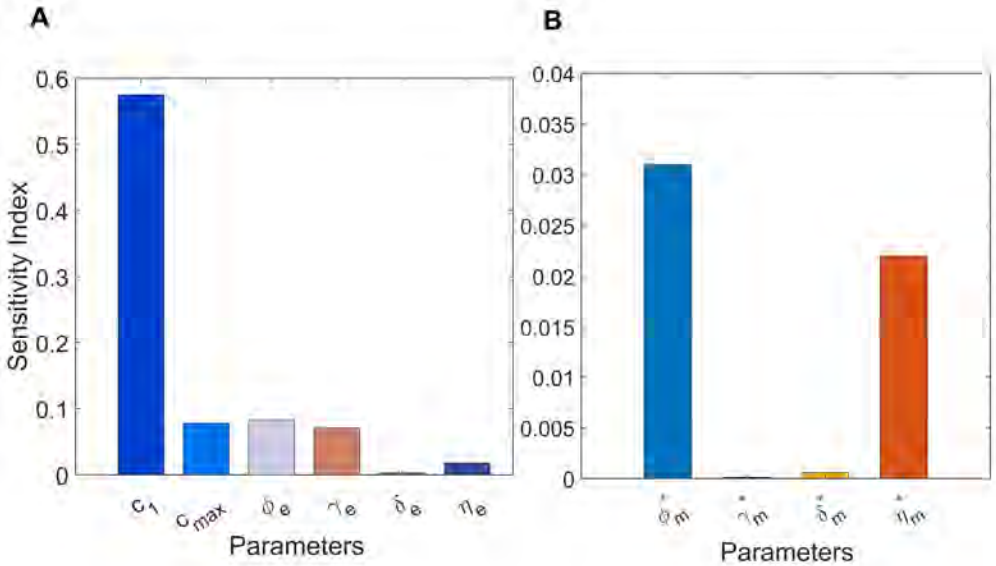


**Fig. S3. A.** Sensitivity analysis in WT mice identifies key parameters influencing model dynamics, with notable sensitivity to $c_1$, $c_{\text{max}}$, $\phi_e$, and $\gamma_e$. **B.** In SIRP$\alpha^{-/-}$ mice, tumor growth is primarily governed by $\phi_m^*$, $\eta_m^*$. While $\delta_m^*$, $\gamma_m^*$ exhibit small sensitivity, they were retained in the model due to the availability of sufficient data and their biological relevance to macrophage-tumor interactions.

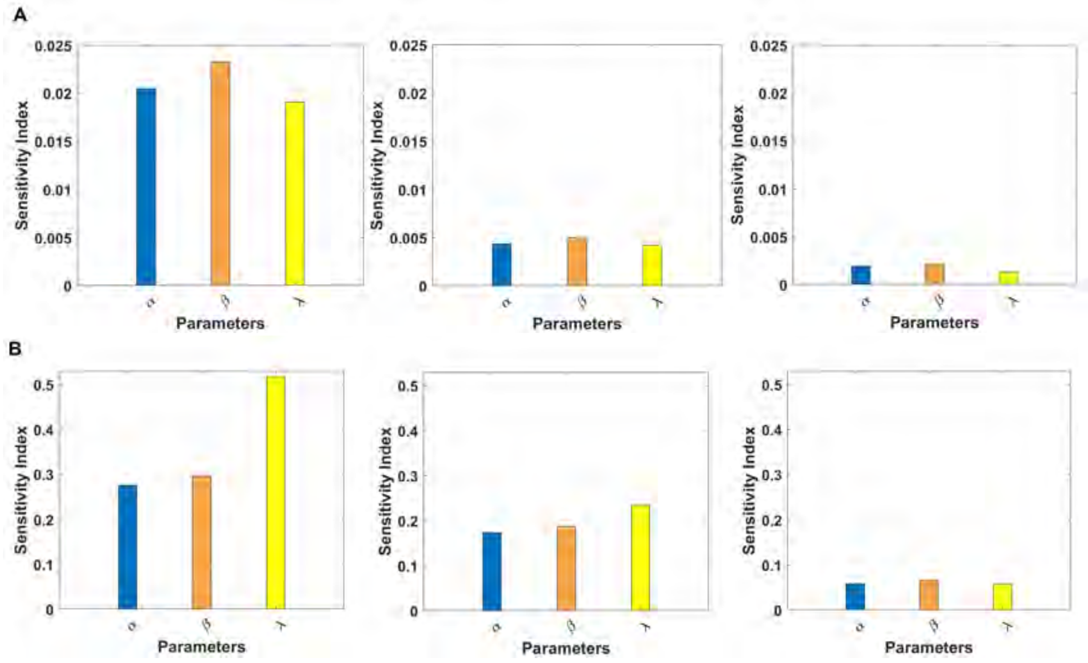


**Fig. S4.** Sensitivity analysis of RT parameters in WT and SIRP$\alpha^{-/-}$ mice across different tumor sizes: **A.** Sensitivity analysis of RT parameters ($\alpha$, $\beta$, and $\lambda$ ) in WT mice for small (left), medium (center), and large (right) tumors. The sensitivity values are in the order of $10^{-3} - 10^{-2}$, indicate minimal influence. **B.** Sensitivity analysis of RT parameters ($\alpha$, $\beta$, and $\lambda$ ) in SIRP$\alpha^{-/-}$ mice for small (left), medium (center), and large (right) tumors. RT parameters $\alpha$, $\beta$, and $\lambda$ indicate greater radiosensitivity in SIRP$\alpha^{-/-}$ mice than in WT mice.

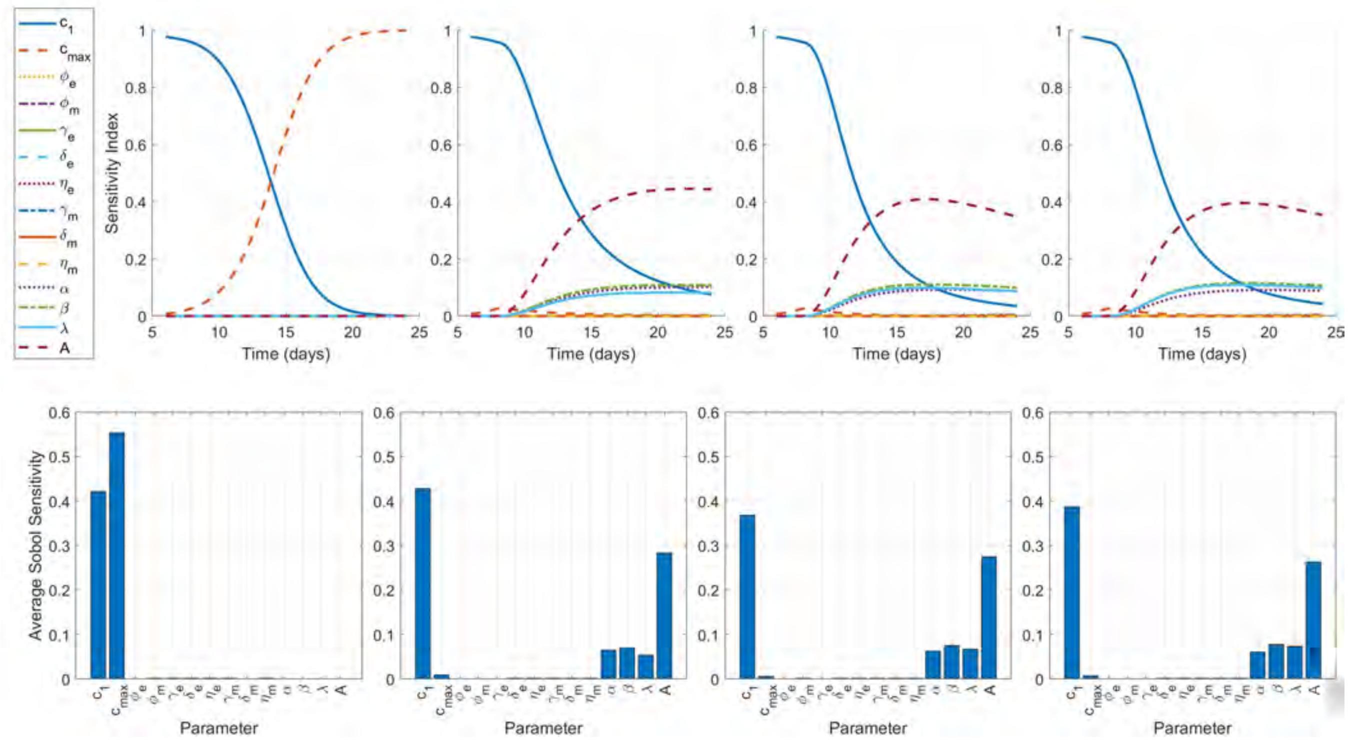

**Fig. S5.** Sobol sensitivity analysis of all parameters in SIRP$\alpha^{-/-}$ mice across different RT doses (no RT, $d = 4$ Gy, $8$ Gy, and $15$ Gy from left to right) highlights $c_1$ and $c_{max}$ as the most sensitive parameters. RT parameters become influential after RT is applied on day 8, gradually dominating tumor dynamics.

 

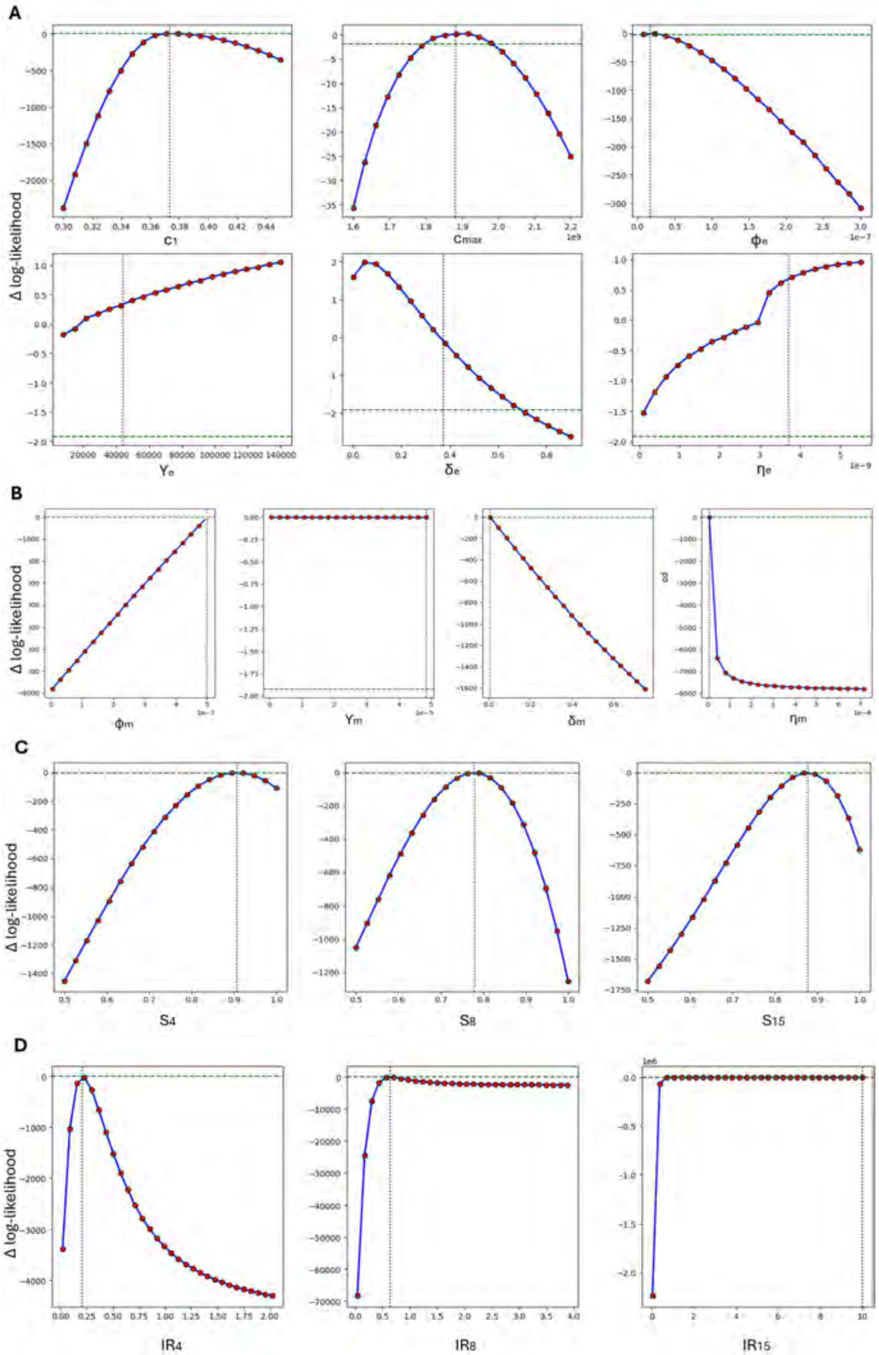


**Fig. S6.** Profile likelihood analyses for different tumor growth models. **A:** WT model, six parameters with three identifiable parameters. **B:** SIRP$\alpha^{-/-}$ model, four parameters are unidentifiable. **C:** Survival function $S$ for tumor growth in WT under RT shows identifiability. **D:** In the radiotherapy case in SIRP$\alpha^{-/-}$ as both the radiotherapy survival fraction and ICD are multiplied by the same factor, so we cannot differentiate them in different terms while doing the noisy data fitting. While on the other hand if we combine these term in a new term irradiation response $IR = A(1-S) - S$ is identifiable for lower and medium RT doses in SIRP$\alpha^{-/-}$ mouse.

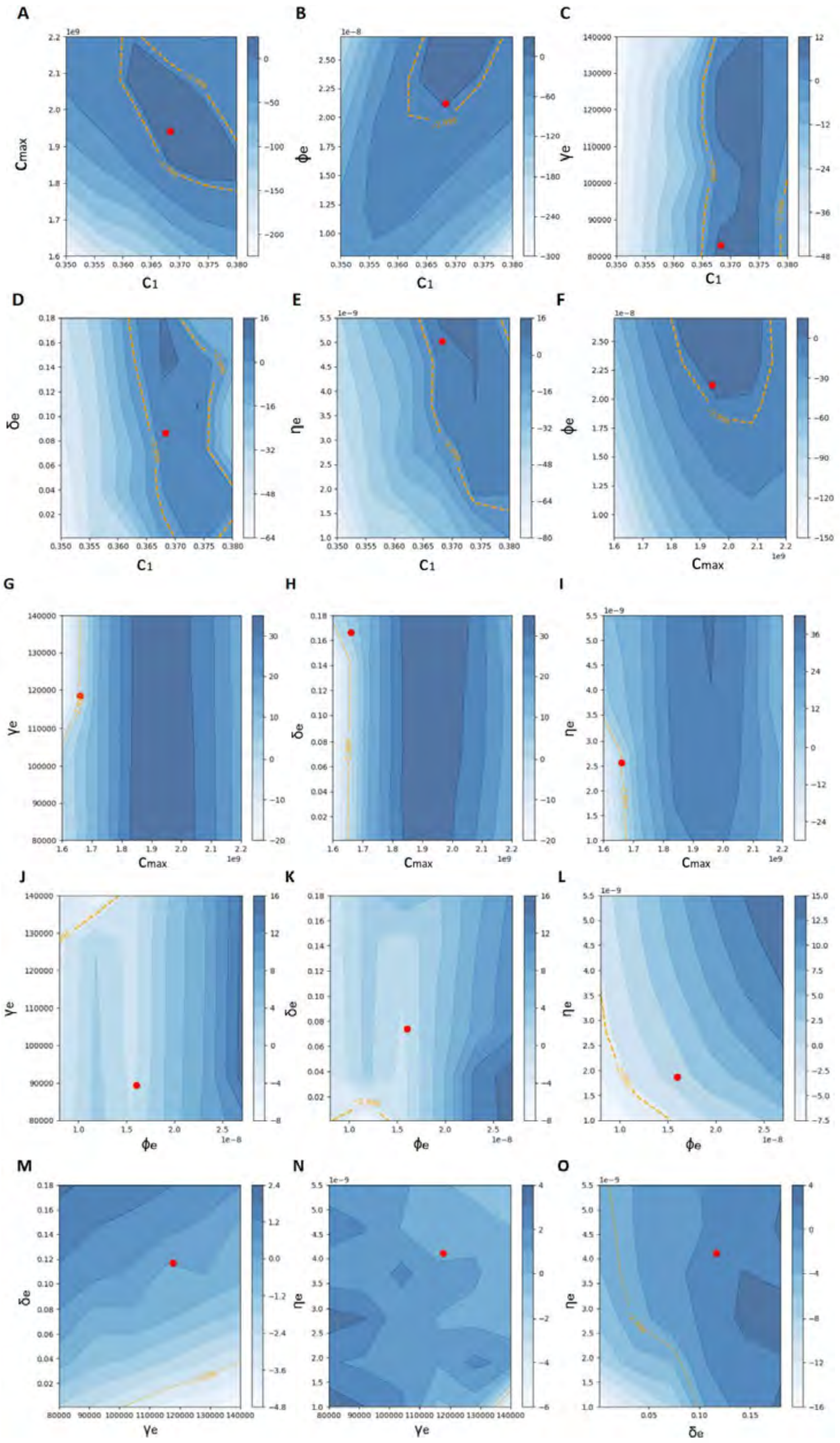


**Fig. S7.** Parameters co-variability for WT model. As $c_1$ and $c_{max}$ are identifiable from the data, we can see that MLE is lying in a closed loop, while for parameters we can see a banana shape or 95%CI region is not bounded, spreading in one direction or the other or both, which means either one or both of the parameters are practically unidentifiable. For $\gamma_e$ and $\delta_e$ we observe a positive correlation while $\phi_e$ and $\eta_e$ are negatively correlated.

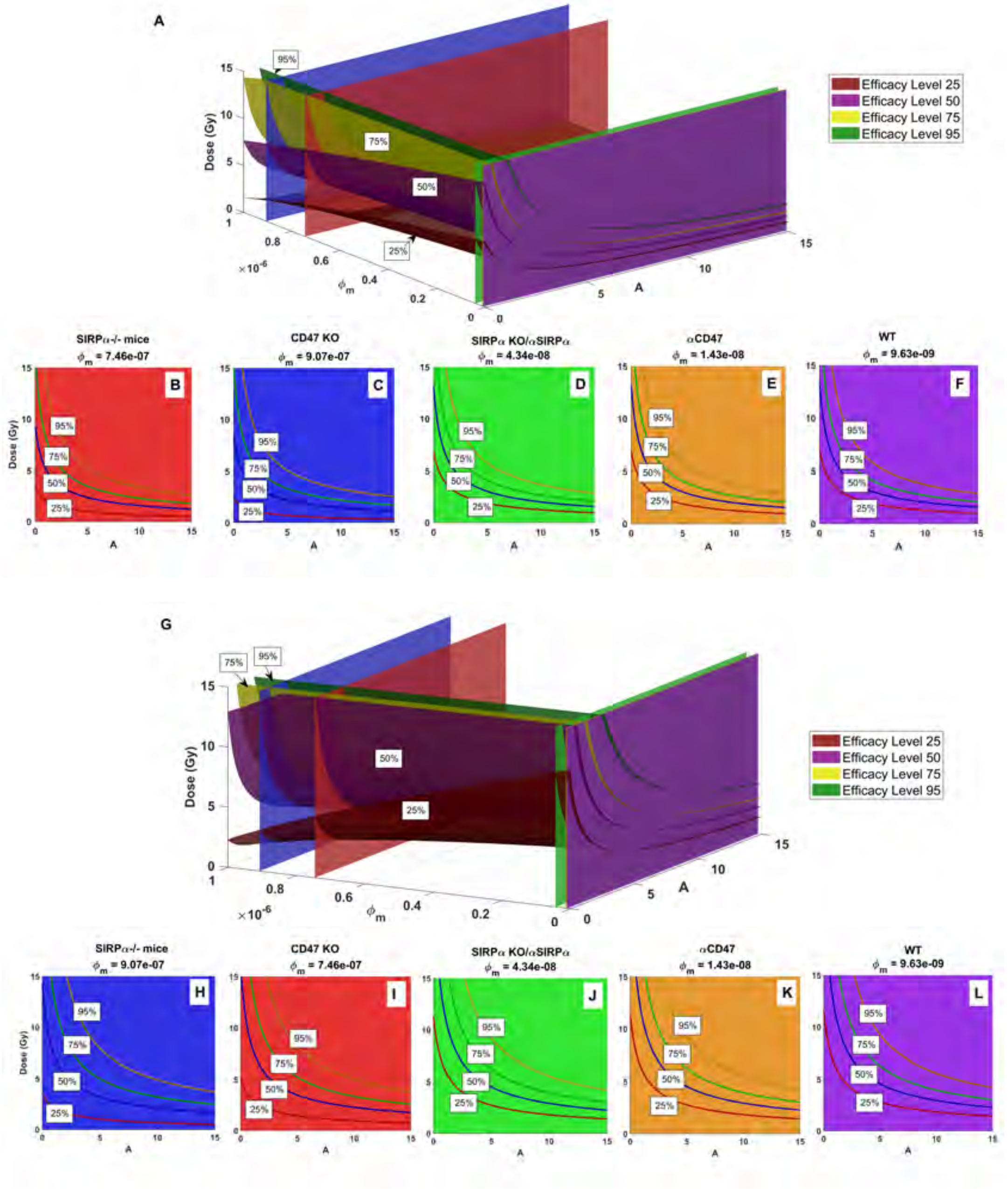


**Fig. S8. Predicted treatment efficacy for combined RT and macrophage-based immunotherapy for small tumors (A-F) and large tumors (G-L):** A &G. Isosurfaces at four efficacy levels (25%, 50%, 75%, and 95%) as a function of ICD activation $A$, macrophage phagocytosis $\phi_m$, and radiation dose $d$. B-F & G-L: Cross-sections of efficacy contours for $\phi_m$ values corresponding to various treatment options for inhibiting the SIRP$\alpha$/CD47 checkpoint.

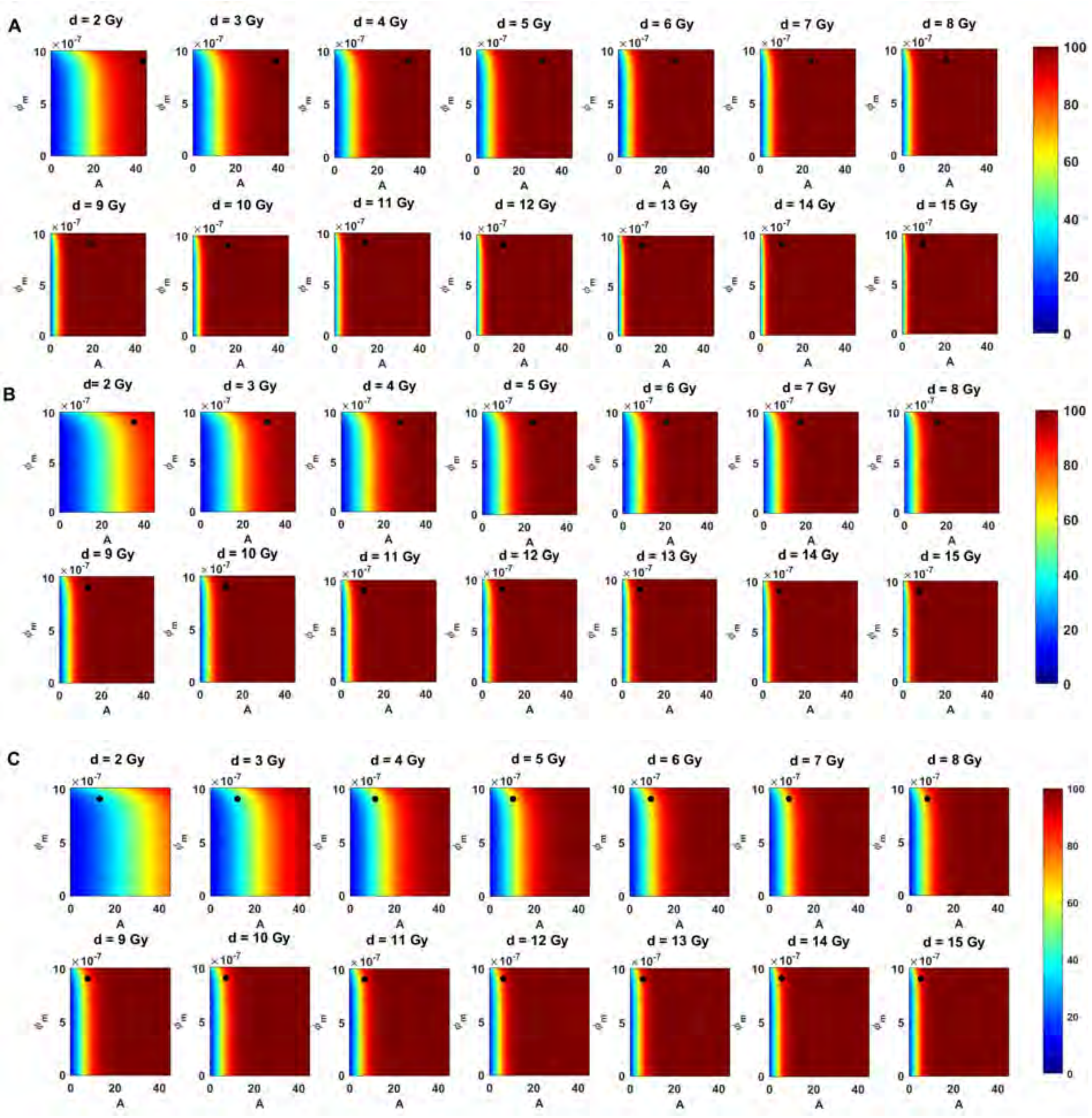


**Fig. S9. Tumor size reduction due to RT:** The heat maps illustrate the predicted impact of radiation dose, phagocytosis, and immune activation on tumor reduction efficacy. **A.** Small tumors exhibit high sensitivity to RT, showing significant reduction across all dose levels. **B.** Medium-sized tumors demonstrate reduced efficacy at lower doses but retain responsiveness at higher doses. **C.** Large tumors display minimal response, particularly at low doses, highlighting the critical role of tumor size and the need for timely intervention to achieve optimal therapeutic outcomes.

**Table S1. Parameter estimation for *in vivo* tumor growth in WT mouse using the CEM model.**

| Parameter (Symbol) | Unit | Literature Value | Fitted Value | 95% CI |
|---|---|---|---|---|
| Tumor growth rate ($c_1$) | $\text{day}^{-1}$ | $4.56 \times 10^{-1}$ | $4.14 \times 10^{-1}$ | [3.5e-1, 6e-1] |
| Tumor carrying capacity ($c_{\max}$) | cell | $2 \times 10^{9}$ | $1.91 \times 10^{9}$ | [1.8e9, 2.1e9] |
| Effector cells killing rate ($\phi_e$) | $(\text{cell} \cdot \text{day})^{-1}$ | $1.0 \times 10^{-7}$ | $3.21 \times 10^{-7}$ | [1e-8, 1e-6] |
| Effector cell influx rate ($\gamma_e$) | $\text{cell} \cdot \text{day}^{-1}$ | $1.3 \times 10^{4}$ | $1.48 \times 10^{4}$ | [1e3, 2.53e4] |
| Effector cell exhaustion rate ($\eta_e$) | $(\text{cell} \cdot \text{day})^{-1}$ | $3.34 \times 10^{-10}$ | $3.31 \times 10^{-10}$ | [1e-10, 1e-9] |
| Effector cell clearance rate ($\delta_e$) | $\text{day}^{-1}$ | $2 \times 10^{-2}$ | $5.9 \times 10^{-2}$ | [2e-3, 2e-1] |
| **Macrophage killing rate ($\phi_m$)** | **$(\text{cell} \cdot \text{day})^{-1}$** | **$6 \times 10^{-9}$** | **$3.57 \times 10^{-10}$** | **[6e-12, 6e-9]** |
| Macrophage influx rate ($\gamma_m$) | $\text{cell} \cdot \text{day}^{-1}$ | – | $3.93 \times 10^{-7}$ | [1e-8, 1e-6] |
| Macrophage exhaustion rate ($\eta_m$) | $(\text{cell} \cdot \text{day})^{-1}$ | $1 \times 10^{-7}$ | $1.77 \times 10^{-7}$ | [1e-8, 1e-6] |
| Macrophage clearance rate ($\delta_m$) | $\text{day}^{-1}$ | $2 \times 10^{-1}$ | $2.17 \times 10^{-2}$ | [2e-3, 8e-1] |

Macrophage influx rate is not available in the literature.

**Table S2. Comparison of tumor growth models in WT mice**

| Model | Equation[a] | Error Rate[b] |
|---|---|---|
| **Generalized Logistic** (19) | $\frac{dC}{dt} = c_1 C^a \left(1 - \left(\frac{C}{c_{\max}}\right)^b\right)^c - \phi_e CE$ | 5.0% |
| **Richards'** (19) | $\frac{dC}{dt} = c_1 C \left(1 - \left(\frac{C}{c_{\max}}\right)^b\right) - \phi_e CE$ | 4.7% |
| **Blumberg's** (19) | $\frac{dC}{dt} = c_1 C^a \left(1 - \frac{C}{c_{\max}}\right)^c - \phi_e CE$ | 4.4% |
| **Logistic** (19) | $\frac{dC}{dt} = c_1 C \left(1 - \frac{C}{c_{\max}}\right) - \phi_e CE$ | 4.3% |
| **Exponential** (19) | $\frac{dC}{dt} = c_1 C - \phi_e CE$ | 11.6% |
| **Gompertz** (19) | $\frac{dC}{dt} = c_1 C \log\left(\frac{c_{\max}}{C}\right) - \phi_e CE$ | 41.4% |
| **Strong Allee Effect** (20) | $\frac{dC}{dt} = c_1 C \left(1 - \frac{C}{c_{\max}}\right) - \phi_e CE \left(\frac{E}{A_l} - 1\right)$ | 4.42% |
| **Weak Allee Effect** (20) | $\frac{dC}{dt} = c_1 C \left(1 - \frac{C}{c_{\max}}\right) - \phi_e CE \left(\frac{E}{A_l} - 1\right)$ | 4.8% |

a. The equation for effector, $\frac{dE}{dt} = \gamma_e - \delta_e E - \eta_e CE$, remains same for all models.

b. Error rate is defined as $||Y_{fit} - Y_{data}||/Y_{data}$.

**Table S3. Parameter estimation for tumor growth in WT mice (CE model)**

| Parameter (Symbol) | Unit | Literature Value | Fitted Value | 95% CI |
|---|---|---|---|---|
| Tumor growth rate ($c_1$) | $day^{-1}$ | $4.56 \times 10^{-1}$ (21) | $4.53 \times 10^{-1}$ | $[3.8e{-1}, 6e{-1}]$ |
| Tumor carrying capacity ($c_{\text{max}}$) | $cell$ | $2 \times 10^9$ (21) | $1.77 \times 10^9$ | $[1.7e9, 2.1e9]$ |
| Effector cells killing rate ($\phi_e$) | $(cell \cdot day)^{-1}$ | $1.0 \times 10^{-7}$ (22) | $4.5 \times 10^{-8}$ | $[2e{-8}, 9.4e{-8}]$ |
| Effector cell influx rate ($\gamma_e$) | $cell \cdot day^{-1}$ | $1.3 \times 10^4$ (5, 22) | $1.65 \times 10^4$ | $[5.0e3, 3.37e4]$ |
| Effector cell exhaustion rate ($\eta_e$) | $(cell \cdot day)^{-1}$ | $3.34 \times 10^{-10}$ (22) | $1.98 \times 10^{-9}$ | $[3.3e{-10}, 3.3e{-9}]$ |
| Effector cell clearance rate ($\delta_e$) | $day^{-1}$ | $2 \times 10^{-2}$ (5) | $7.41 \times 10^{-2}$ | $[6.8e{-3}, 2e{-1}]$ |

**Table S4. Parameter estimation for macrophage in SIRP$\alpha^{-/-}$ mice.**

| Parameter (Symbol) | Unit | Literature Value for M1 macrophage | Fitted Value | 95% CI |
|---|---|---|---|---|
| $M^*$ killing rate ($\phi_m^*$) | $(cell \cdot day)^{-1}$ | $6 \times 10^{-9}$(10) | $9.07 \times 10^{-7}$ | $[1e{-7}, 1e{-5}]$ |
| $M^*$ influx rate ($\gamma_m^*$) | $cell \cdot day^{-1}$ | – | $4.39 \times 10^{-7}$ | $[1e{-8}, 1e{-6}]$ |
| $M^*$ exhaustion rate ($\eta_m^*$) | $(cell \cdot day)^{-1}$ | $1 \times 10^{-7}$(10) | $8.15 \times 10^{-7}$ | $[1e{-8}, 1e{-6}]$ |
| $M^*$ clearance rate ($\delta_m^*$) | $day^{-1}$ | $2 \times 10^{-1}$(21) | $3.94 \times 10^{-1}$ | $[2e{-3}, 8e{-1}]$ |

**Table S5. Parameters for Linear Quadratic Radiotherapy Model**

| Parameter (Symbol) | Unit | Literature Value | Mean of Fitted Values | 95% CI |
|---|---|---|---|---|
| Linear damage coefficient ($\alpha$) | $Gy^{-1}$ | $2.8 \times 10^{-8}$ (13) | $5.15 \times 10^{-3}$ | [0, $5.4e{-}3$] |
| Quadratic damage coefficient ($\beta$) | $Gy^{-2}$ | $1.32 \times 10^{-2}$ (13) | $6.58 \times 10^{-3}$ | [0, $2.24e{-}2$] |
| Repair parameter ($\lambda$) | $day^{-1}$ | $2.036$ (13) | 2.56 | [$2.54$, 5.00] |

**Table S6. Comparison of parameter values across cell lines and mouse models**

| **Cell Line/Model [data source]** | $\boldsymbol{c_1}$ $(\times 10^{-1})$ | $\boldsymbol{c}_{\text{max}}$ $(\times 10^{9})$ | $\boldsymbol{\alpha}$ $(\times 10^{-3})$ | $\boldsymbol{\beta}$ $(\times 10^{-3})$ |
|---|---|---|---|---|
| MC38 in WT (1) | $4.53$ | $1.77$ | $5.15$ | $6.58$ |
| Pan02 in WT (1) | $4.40$ | $2.00$ | $5.15$ | $6.58$ |
| KPC in WT (1) | $4.80$ | $2.00$ | $5.15$ | $6.58$ |
| Fibrosarcoma in WT BALB/c (15) | $3.59$ | $4.50$ | $5.15$ | $6.58$ |
| NSG KP1 SCLC (2) | $5.10$ | $4.08$ | $3.80 \times 10^{-6}$ | $25.8$ |
| MC38-OVA in WT (3) | $4.53$ | $1.77$ | $1.00$ | $65.0$ |
| MC38 in WT (4) | $1.60$ | $4.32$ | $5.15$ | $6.58$ |

**Table S7. Overview of experimental setups and modeling parameters across different treatments, simultaneous datasets fitting without bootstrap.**

| mouse model | Cell Line | Description | Data Points | Fitted Parameters | Remarks |
|---|---|---|---|---|---|
| C57BL/6 (WT) | MC38 | Mice were s.c. engrafted with four initial injections | 32 | $c_1 = 4.53 \times 10^{-1}$, $c_{\max} = 1.77 \times 10^{9}$, $\phi_e = 4.5 \times 10^{-8}$, $\gamma_e = 1.65 \times 10^{4}$, $\eta_e = 1.98 \times 10^{-9}$, $\delta_e = 7.41 \times 10^{-2}$ | Parameters remain within the same orders of magnitude reported in literature, 7% error. |
| SIRP$\alpha^{-/-}$ | MC38 | Mice were s.c. engrafted with four injections | 32 | $\phi^*_m = 9.07 \times 10^{-7}$, $\gamma^*_m = 4.39 \times 10^{-7}$, $\eta^*_m = 8.15 \times 10^{-7}$, $\delta^*_m = 3.94 \times 10^{-1}$ | Error is 7%, the parameters fitted in WT mice remain constant. |
| WT with RT | MC38 | Mice were s.c. engrafted with $5 \times 10^5$/mouse of MC38 cells, with control, 4, 8, and 15 Gy RT applied to small, medium, and large tumor | D.1: 35, D.2: 30, D.3: 29 | $\alpha = 5.15 \times 10^{-3}$, $\beta = 6.58 \times 10^{-3}$, $\lambda = 2.56$ | The parameters fitted in WT mice remain constant. The error rates are 8%, 5%, and 8%. |
| SIRP$\alpha^{-/-}$ with RT | MC38 | Mice were s.c. engrafted with $5 \times 10^5$/mouse of MC38 cells, and no RT, 4, 8, and 15 Gy radiation treatments were applied on days 8, 12, and 14 | D.1: 37, D.2: 37, D.3: 34 | $A = 34.9, 21.1, 9.6$, $A = 27.7, 15.3, 7.7$, $A = 11.3, 8.2, 5.3$ | The error rates are 5%, 8%, and 6%. The parameters fitted in WT mice and WT RT remain constant. |
| SIRP$\alpha^{-/-}$ with RT | MC38 | Disrupting SIRP$\alpha$-deficient macrophages using Cl2MDA liposomes or an antibody against the CSF1 receptor ($\alpha$CSF1R) from SIRP$\alpha^{-/-}$ mice, then 8 Gy RT was applied on day 12 | 36 | All previous values used | All other parameters remain constant. |
| WT with RT | MC38 | The i.v. injection of SIRP$\alpha$-deficient macrophages into WT, then 8 Gy RT was applied on days 12 and 14 | 18 | $A_{d1} = 8.247$, $A_{d2} = 19.95$ and $\phi^*_m = 4.43 \times 10^{-8}$ | All other parameters remained constant. |
| WT with RT | MC38 | The i.t. injection of BMDM and SIRP$\alpha^{-}$ macrophages into WT, 8 Gy RT applied on day 12 | 34 | $A$ values from i.v. case are used. 7.2% error rate is reported. All parameters remain constant. | |
| WT with RT | MC38 | The i.t. injection of BMDM and SIRP$\alpha^{-}$ macrophages into WT, 8 Gy RT applied on days 12 and 14 | 34 | $A$ values from i.v. case are used. 9.9% error rate is observed. All parameters remain constant. | |
| WT+SIRP$\alpha^{-/-}$ with RT | KPC | (s.c.) injected KPC into WT and SIRP$\alpha^{-/-}$, 8 Gy RT applied on day 18 | 31 | $c_1 = 4.8 \times 10^{-1}$, $c_{max} = 2 \times 10^{9}$, $A = 17.24$ | 4.9% error rate. All other parameters remain constant. |
| WT+SIRP$\alpha^{-/-}$ with RT | Pan02 | (s.c.) injected Pan02 into WT and SIRP$\alpha^{-/-}$, 8 Gy RT applied on day 12 | 32 | $c_1 = 4.4 \times 10^{-1}$, $c_{max} = 2 \times 10^{9}$, $A = 15.33$ | 3.9% error rate. All other parameters remain constant. |
| WT BALB/c Mice with RT | 15-12RM Fibrosarcoma | Intraperitoneally (i.p.) injected into Athymic BALB/c nu/nu mice with saline as a control or with a solution of 10 mmol/L CD47 morpholino (CD47M) in saline with 10 Gy RT applied on day 10 | 27 | $c_1 = 3.59 \times 10^{-1}$, $c_{max} = 4.5 \times 10^{9}$, $A = 0$ | 8.3% error rate is measured. All other parameters remain constant. |

Continued on next page

| mouse model | Cell Line | Description | Data Points | Fitted Parameters | Remarks |
|---|---|---|---|---|---|
| Immunodeficient NSG mice with RT | KP1 SCLC | Anti-CD47 applied. Two datasets with a 5 Gy dose applied on days 10 or 12 | D.1: 20, D.2: 20 | $c_1 = 5.1 \times 10^{-1}$, $c_{max} = 4.08 \times 10^9$, $\alpha = 3.8 \times 10^{-9}$, $\beta = 2.58 \times 10^{-2}$, $\phi_m^* = 1.43 \times 10^{-8}$, $A = 1.42$, $A = 5.41$ | 4.3% and 11.8% error rates are observed. All other parameters remain constant. |
| Immunodeficient NSG mice with RT | KP1 SCLC | CD47-KO for two datasets of RT 5 Gy double doses applied on days 10, 14, 11, and 13: D.1: 22, D.2: 21 | $A_{d1} = 0.16$, $A_{d2} = 2.47$, $A_{d1} = 0.97$, $A_{d2} = 2.97$ | 14.6% and 15.2% error rates. All other parameters remain constant on the basis of anti-CD47 fitting. | |
| WT Mice with RT | MC38-OVA | anti-CD47/anti-SIRP$\alpha$ treatment with 8 Gy applied on days 12 | 32 | $\alpha = 1 \times 10^{-3}$, $\beta = 6.5 \times 10^{-2}$,$\phi_m^* = 4.34 \times 10^{-8}$,$A_{cd} = 0.27$, and $A_{sa} = 0.33$ | 10.6% error; RT delivery time was not given in the article; we assumed 6 Gy/min. All other parameters remain constant on the basis of anti-CD47 fitting. |
| WT mice with RT | MC38 | Anti-SIRP$\alpha$ treatment with 12 Gy RT applied on day 9 | 32 | $c_1 = 1.6 \times 10^{-1}$, $c_{max} = 4.32 \times 10^9$ and $A = 0.99$ | 9.3% error; RT delivery time not given in the article; we assumed 6 Gy/min. All other parameters remain constant on the basis of anti-SIRP$\alpha$ fitting in WT mice. The reason for fitting $c_1$ and $c_{max}$ is to reduce tumor growth in this study. |

## References


1. Z Bian, et al., Intratumoral sirp$\alpha$-deficient macrophages activate tumor antigen-specific cytotoxic t cells under radiotherapy. *Nat. communications* **12**, 3229 (2021).
2. Y Nishiga, et al., Radiotherapy in combination with cd47 blockade elicits a macrophage-mediated abscopal effect. *Nat. Cancer* **3**, 1351–1366 (2022).
3. RCE Hsieh, et al., Atr-mediated cd47 and pd-l1 up-regulation restricts radiotherapy-induced immune priming and abscopal responses in colorectal cancer. *Sci. immunology* **7**, eabl9330 (2022).
4. K Ji, et al., Sirp$\alpha$ blockade improves the antitumor immunity of radiotherapy in colorectal cancer. *Cell Death Discov.* **9**, 180 (2023).
5. LG de Pillis, AE Radunskaya, CL Wiseman, A validated mathematical model of cell-mediated immune response to tumor growth. *Cancer research* **65**, 7950–7958 (2005).
6. D Kirschner, JC Panetta, Modeling immunotherapy of the tumor–immune interaction. *J. mathematical biology* **37**, 235–252 (1998).
7. RA Bekker, S Kim, S Pilon-Thomas, H Enderling, Mathematical modeling of radiotherapy and its impact on tumor interactions with the immune system. *Neoplasia* **28**, 100796 (2022).
8. B Hoffmann, et al., The initial engraftment of tumor cells is critical for the future growth pattern: a mathematical study based on simulations and animal experiments. *BMC cancer* **20**, 1–14 (2020).
9. L Monteiro, M Rodrigues, D Gomes, B Salgado, G Cassali, Tumour-associated macrophages: Relation with progression and invasiveness, and assessment of m1/m2 macrophages in canine mammary tumours. *The Vet. J.* **234**, 119–125 (2018).
10. R Eftimie, H Hamam, Modelling and investigation of the cd4+ t cells–macrophages paradox in melanoma immunotherapies. *J. theoretical biology* **420**, 82–104 (2017).
11. Y Murata, Y Saito, T Kotani, T Matozaki, Cd 47-signal regulatory protein $\alpha$ signaling system and its application to cancer immunotherapy. *Cancer Sci.* **109**, 2349–2357 (2018).
12. R Klaus, M Niyazi, B Lange-Sperandio, Radiation-induced kidney toxicity: molecular and cellular pathogenesis. *Radiat. oncology* **16**, 43 (2021).
13. J Poleszczuk, H Enderling, The optimal radiation dose to induce robust systemic anti-tumor immunity. *Int. journal molecular sciences* **19**, 3377 (2018).
14. R Hawkins, Effect of heterogeneous radio sensitivity on the survival, alpha beta ratio and biologic effective dose calculation of irradiated mammalian cell populations. *Clin. translational radiation oncology* **4**, 32–38 (2017).
15. DR Soto-Pantoja, et al., Cd47 in the tumor microenvironment limits cooperation between antitumor t-cell immunity and radiotherapy. *Cancer research* **74**, 6771–6783 (2014).
16. S Pan, et al., Radiation exposure–induced changes in the immune cells and immune factors of mice with or without primary lung tumor. *Dose-Response* **18**, 1559325820926744 (2020).
17. KI Gasior, NG Cogan, Untangling the molecular interactions underlying intracellular phase separation using combined global sensitivity analyses. *Bull. Math. Biol.* **86**, 60 (2024).
18. P Jain, et al., Cell-state transitions and density-dependent interactions together explain the dynamics of spontaneous epithelial-mesenchymal heterogeneity. *Iscience* **27** (2024).
19. B Cioruţa, M Coman, Aspects regarding the limited population growth models characterized by the logistic equation applets. *Int. Multidiscip. Sci. GeoConference: SGEM* **1**, 429–436 (2016).
20. KE Johnson, et al., Cancer cell population growth kinetics at low densities deviate from the exponential growth model and suggest an allee effect. *PLoS biology* **17**, e3000399 (2019).
21. Y Shu, J Huang, Y Dong, Y Takeuchi, Mathematical modeling and bifurcation analysis of pro-and anti-tumor macrophages. *Appl. Math. Model.* **88**, 758–773 (2020).
22. VA Kuznetsov, IA Makalkin, MA Taylor, AS Perelson, Nonlinear dynamics of immunogenic tumors: parameter estimation and global bifurcation analysis. *Bull. mathematical biology* **56**, 295–321 (1994).
